# HIV-1 and SIV Predominantly Use CCR5 Expressed on a Precursor Population to Establish Infection in T Follicular Helper Cells

**DOI:** 10.3389/fimmu.2017.00376

**Published:** 2017-04-21

**Authors:** Yin Xu, Chansavath Phetsouphanh, Kazuo Suzuki, Anu Aggrawal, Stephanie Graff-Dubois, Michael Roche, Michelle Bailey, Sheilajen Alcantara, Kieran Cashin, Rahuram Sivasubramaniam, Kersten K. Koelsch, Brigitte Autran, Richard Harvey, Paul R. Gorry, Arnaud Moris, David A. Cooper, Stuart Turville, Stephen J. Kent, Anthony D. Kelleher, John Zaunders

**Affiliations:** ^1^The Kirby Institute, The University of New South Wales, Sydney, NSW, Australia; ^2^St Vincent’s Centre for Applied Medical Research, St Vincent’s Hospital, Sydney, NSW, Australia; ^3^Sorbonne Universités, UPMC Univ Paris 06, INSERM U1135, CNRS ERL 8255, Center for Immunology and Microbial Infections – CIMI-Paris, Paris, France; ^4^Department of Microbiology and Immunology, Peter Doherty Institute, University of Melbourne, Melbourne, VIC, Australia; ^5^Center for Biomedical Research, Burnet Institute, Melbourne, VIC, Australia; ^6^Sorbonne Universités, UPMC University Paris 06, INSERM U1135, Center for Immunology and Microbial Infections – CIMI-Paris, Paris, France; ^7^AP-HP, Hôpital Pitié-Salpêtière, Department of Immunology, Paris, France; ^8^School of Health and Biomedical Sciences, College of Science, Engineering and Health, RMIT University, Bundoora, VIC, Australia; ^9^Department of Infectious Diseases, Alfred Hospital, Monash University, Melbourne, VIC, Australia

**Keywords:** HIV-1, CD4, T follicular helper cells, germinal center, CCR5

## Abstract

**Background:**

T follicular helper (Tfh) cells are increasingly recognized as a major reservoir of HIV infection that will likely need to be addressed in approaches to curing HIV. However, Tfh express minimal CCR5, the major coreceptor for HIV-1, and the mechanism by which they are infected is unclear. We have previously shown that macaque Tfh lack CCR5, but are infected *in vivo* with CCR5-using SIV at levels comparable to other memory CD4^+^ T cells. Similarly, human splenic Tfh cells are highly infected with HIV-1 DNA. Therefore, we set out to examine the mechanism of infection of Tfh cells.

**Methodology:**

Tfh and other CD4^+^ T cell subsets from macaque lymph nodes and spleens, splenic Tfh from HIV^+^ subjects, and tonsillar Tfh from HIV-uninfected subjects were isolated by cell sorting prior to cell surface and molecular characterization. HIV proviral gp120 sequences were submitted to genotypic and phenotypic tropism assays. Entry of CCR5- and CXCR4-using viruses into Tfh from uninfected tonsillar tissue was measured using a fusion assay.

**Results:**

Phylogenetic analysis, genotypic, and phenotypic analysis showed that splenic Tfh cells from chronic HIV^+^ subjects were predominantly infected with CCR5-using viruses. In macaques, purified CCR5^+^PD-1^intermediate(int)+^ memory CD4^+^ T cells were shown to include pre-Tfh cells capable of differentiating *in vitro* to Tfh by upregulation of PD-1 and Bcl6, confirmed by qRT-PCR and single-cell multiplex PCR. Infected PD-1^int^ cells survive, carry SIV provirus, and differentiate into PD-1^hi^ Tfh after T cell receptor stimulation, suggesting a pathway for SIV infection of Tfh. In addition, a small subset of macaque and human PD-1^hi^ Tfh can express low levels of CCR5, which makes them susceptible to infection. Fusion assays demonstrated CCR5-using HIV-1 entry into CCR5^+^ Tfh and pre-Tfh cells from human tonsils.

**Conclusion:**

The major route of infection of Tfh in macaques and humans appears to be *via* a CCR5-expressing pre-Tfh population. As the generation of Tfh are important for establishing effective immune responses during primary infections, Tfh are likely to be an early target of HIV-1 following transmission, creating an important component of the reservoir that has the potential to expand over time.

## Introduction

T follicular helper (Tfh) cells are a subset of memory CD4^+^ T cells located in secondary lymphoid tissues such as tonsil, spleen, and lymph node (LN). The process of Tfh cell differentiation is multistage, multifactorial, and also involves migration within the microenvironment of secondary lymphoid tissue. In the T cell area, naïve CD4^+^ T cells are primed by dendritic cells (DC) and differentiate toward a CXCR5^+^ICOS^+^Bcl6^+^ Tfh cell intermediate (1), accompanied by the upregulation of PD-1 and downregulation of CCR7 (2). These DC primed CD4^+^ T cells, with their increased expression of Bcl6 and CXCR5, are able to migrate to the interfollicular area, also known as the T:B border, where they encounter B cells (3). Interaction with cognate B cells *via* ICOS and ICOS ligand interaction is critical for Tfh cell commitment, as initial Tfh cell phenotypes obtained at the DC priming stage are lost during further rounds of division in the absence of B cells (4). Tfh cells that have successful interactions with cognate B cells migrate inside the follicle to complete full differentiation and to support the germinal center (GC) reaction (5, 6). This process requires further increases in Bcl6 expression and several changes in chemokine receptor expression, allowing proper localization into GC as well as high-level expression of adhesion molecules to stabilize cognate T–B interaction. Tfh cells at this stage are sometimes called GC Tfh cells to distinguish them from those located at the T:B border, and those in the follicle but outside GC (5). Functionally, Tfh cells secrete IL-21, IL-4, and/or IFN-γ (5, 7). IL-21, in conjunction with costimulatory signals including CD40–CD40L interaction, drives proliferation, and differentiation of B cells (8–10). It also acts on Tfh cells in an autocrine manner to promote Tfh cell differentiation, although this effect is redundant with IL-6 (11).

The expression of surface protein PD-1 has been used in multiple studies to define Tfh cells in lymphoid tissue in humans and macaques, usually in combination with other surface markers such as CXCR5 or CD127 (12–14). This is because PD-1, particularly when stained with the monoclonal antibody EH12 clone, clearly separates the memory CD4^+^ T cells into PD-1^low(lo)^, PD-1^intermediate(int)^, and PD-1^high(hi)^ populations (12–14). The distinct PD-1^hi^ population has been shown to express the highest levels of Tfh cell-associated markers CXCR5, IL-21, and Bcl6 (12–14). Immunofluorescent staining of lymphoid tissues demonstrates that PD-1 intensity correlates with the distance of the cell to the center of the GC: the closer to the GC center, the higher PD-1 expression on the cells (15, 16).

It has been reported in both humans and macaques that PD-1^hi^ Tfh cells are infected with HIV-1 or pathogenic SIV at high levels (12–15). However, the literature suggests that Tfh cells express the cell surface chemokine receptor CXCR4, but not CCR5 (5, 17) though, a recent study suggests that up to 30% of human Tfh may be CCR5^+^ (18). We have previously shown that the proviral DNA sequences in Tfh from SIV-infected macaques are predominantly CCR5 tropic (14). The mechanism by which CCR5-tropic SIV is present at high levels in PD-1^hi^ Tfh cells in macaques has not been defined.

SIV infection of Tfh occurs from early in the course of infection and does not wane over the course of the disease (14). Although, extensive longitudinal studies have not been carried out in humans, cross sectional studies suggest a similar temporal profile of infection (13). Furthermore, there is increasing evidence that follicular hyperplasia does not completely resolve following antiretroviral therapy (ART) (19), and that the preferential carriage of HIV-1 in Tfh persists following therapy (20). These cells therefore represent a substantial and unexpected component of the residual reservoir in ART-treated patients.

Given the multiple roles of Tfh cells in immunodeficiency virus infection—as targets and critical helpers in antibody responses, it is important to understand how this component of the therapy-resistant reservoir is established in order to develop rational therapies to limit the HIV-1 reservoir. We therefore set out to explain how this predominantly CCR5-negative population of T cells becomes infected by the CCR5-tropic form of the virus, by conducting a series of parallel experiments in lymphoid tissue cells from both humans and macaques. Our results suggest that while there may be some direct infection of CCR5^+^ Tfh by SIV or HIV-1, the majority of infection most likely occurs at a CCR5^+^ pre-Tfh stage of differentiation.

## Materials and Methods

### Study Subjects, Ethics, and Tissue Processing

Splenic mononuclear single-cell suspensions were obtained and viably cryopreserved from three chronic HIV-infected patients with idiopathic thrombocytopenic purpura, who required therapeutic splenectomy (21–23). These samples were collected in the early 1990s and plasma viral load estimations are not available. Sample collection followed national ethical guidelines in France regulating the use of human tissues.

Spleen and LN samples were taken from 14 SIV-infected pigtail macaques (Table S1 in Supplementary Material). Inguinal LN was also taken from 15 uninfected macaques. All experiments involving pigtail macaques were approved by the Animal Ethics Committee of the Australian Animal Health Laboratory, Geelong, Victoria, CSIRO Livestock Industries. Single-cell suspensions were prepared from macaque spleens and LN as previously described (14).

Tonsil samples were taken from HIV-uninfected patients undergoing tonsillectomy for other clinical purposes. Participants provided written, informed consent and these studies were approved by the Human Research Ethics Committees of St Vincent’s Hospital, Sydney (HREC 12/077) and The University of New South Wales, Australia (HREC HC12582). Single-cell suspensions were prepared from human tonsils using the same methodology employed for macaque LN (14).

### Flow Cytometry

Monoclonal antibodies (mAb) to human or non-human primate proteins were CD3-Alexa Fluor (AF) 700, -PE-Cy7, -Pacific Blue (PB), -Brilliant Violet (BV) 421, -PE-Cy7, or -APC (clone UCHT1 or SP34-2), CD4-AF700 or -PerCP-Cy5.5 (clone RPA-T4 or L200), CD8-APC-Cy7, -AF700 or -PB (clone SK1 or RPA-T8), CD19-PE (clone HIB19), CD45RA-APC, -AF700 or -PE-CF594 (clone HI100), CXCR5-AF647 or -PerCP-Cy5.5 (clone RF8B2), CCR5-PE or purified (clone 2D7 or 3A9), and Bcl6-PE or -AF647 (clone K112-91) from BD Biosciences (San Jose, CA, USA); ICOS-PE (clone C398.4A), PD-1-PE, -AF647 or -BV421 (clone EH12.2H7), CD127-BV421 (clone A019D5), and FoxP3-AF488 (clone 259D) from Biolegend. CD45RA-ECD (clone 2H4) from Beckman Coulter (Hialeah, FL, USA); CXCR5-APC (clone MU5UBEE) from eBioscience. PE-conjugated AffiniPure F(ab′)2 fragment goat anti-mouse IgG (H+L) secondary antibody from Jackson ImmunoResearch Laboratories (West Grove, PA, USA).

Splenic Tfh were isolated by cell sorting from HIV^+^ subjects as previously described (23).

Labeling cells with molecular probes, detection of surface markers and optimal detection of CCR5, detection of intracellular proteins, such as the active form of Caspase-3, and detection of the intranuclear transcription factors (TFs) such as Bcl6 were performed as previously described (14). Immunophenotyping samples were analyzed on LSRII analyzer or sorted on FACSAria IIu sorter using FACSDiva v5 or v6 software (Becton Dickinson). Data were recorded and further analyzed using FlowJo v8 (TreeStar Inc., OR, USA).

### Quantification of Proviral DNA Levels

Total HIV-1 DNA levels were quantified by qPCR using primers mf299 and mf302, and probes ri15 and ri16, which target the *pol* gene (24). HIV-1 DNA copy numbers were normalized for total cellular DNA input using the TaqMan^®^ β-actin detection kit (Life Technologies, CA, USA). SIV proviral DNA was quantified by qPCR with primers *SIVgag*-F and *SIVgag*-R and locked nucleic acid (LNA) probe *SIVgag*-P that target a highly conserved region of SIV-*gag* p17 matrix and normalized to macaque β-actin (14). Sequences for primers and probes are listed in Table S2 in Supplementary Material.

### Detection of Macaque TF Genes in Single Cells by Multiplex PCR and Quantification in Bulk Population by Conventional RT-qPCR

Transcription factor genes *tbx21, gata3, rorc, bcl6*, and *foxp3*, as well as the housekeeping gene *actb*, were detected in single cells by two-step multiplex PCR, or quantified in bulk populations by conventional RT-qPCR. Primers and LNA probes were adapted from Ref. (25) and modified specifically for macaques. Sequences for primers and probes are listed in Table S2 in Supplementary Material.

For multiplex qPCR, cells were sorted at 1 cell per well into 10 µl of reaction mix in 96-well PCR plates. Single cells were sorted into 94 wells of the plate. The A1 well served as negative control and the H12 well was loaded with total RNA extracted from bulk CD4^+^ T cells and served as positive control. Each reaction mix contained buffer and enzymes from the SuperScript^®^ III One-step RT-PCR System with Platinum^®^ *Taq* DNA polymerase (Invitrogen, Life Technologies), RNase inhibitor (Ambion, Life Technologies) and primer mix containing forward and reverse primers for *actb* (25 nM), *bcl6* (25 nM), *tbx21* (30 nM), *gata3* (30 nM), *rorc* (30 nM), and *foxp3* (40 nM). Cycling conditions for the first round RT and pre-amplification were 1 cycle of 50°C for 30 min, 1 cycle of 95°C for 2 min, and 22 cycles of 95°C for 15 s and 60°C for 4 min. The first round products were diluted 1 in 5 with DNase-free water. The 10 µl second round reaction mix contained 1× LightCycler^®^ 480 Probes Master (Roche, Basel, Switzerland), 1 µM of primers and 0.5 µM of probe for each gene, and 2 µl of diluted first round product. The second round reaction was performed on LightCycler^®^ 480 system with the following cycling conditions: 1 cycle of 95°C for 5 min and 45 cycles of 95°C for 15 s, 60°C for 50 s, and 72°C for 1 s.

Expression of macaque or human TF genes was quantified by two-step RT-qPC from bulk populations. RT was performed using SuperScript™ III First-Strand Synthesis SuperMix for qRT-PCR (Invitrogen) as per manufacturer’s instructions. qPCR was performed using LightCycler^®^ 480 Probes Master (Roche Applied Science) as described above for second round.

### Quantification of *ccr5* in Bulk Population by Conventional RT-qPCR

*ccr5* mRNA levels were quantified by qPCR using primers *ccr5*-F and *ccr5*-R (refer to Table S2 in Supplementary Material for oligonucleotide sequences). Amplification was performed using SYBR Green I Master (Roche, Basel, Switzerland) on a LightCycler^®^ 480 System (Roche Applied Science) as previously described (14).

### Clonal Sequencing of HIV-1 Envelope gp120

The gp120 region of HIV-1 was amplified from gDNA extracted from purified CD4^+^ T cell subsets by nested PCR using Platinum^®^ *Taq* DNA polymerase High Fidelity (Life Technologies, Grand Island, NY, USA). Each 20 µl reaction contained 1× High Fidelity PCR Buffer (minus Mg), 0.2 mM dNTP, 2 mM MgSO_4_, 0.5 U Platinum^®^ *Taq* DNA Polymerase High Fidelity, Nuclease-free water, 0.2 µM each primer set, and template DNA. Cycling conditions were 1 cycle at 94°C for 2 min; 45 cycles of 94°C for 15 s, 55°C for 30 s, and 68°C for 2 min; and 1 cycle at 68°C for 10 min. The first round primers E00F and CO602N target the outer boundaries of the gp120 sequence (nucleotides 6,208–7,817 of HXB2 genome). The second round primers E20F and CD4R target the regions upstream of V1 and downstream of V5, respectively (nucleotides 6,437–7,675 of HXB2 genome) (sequences for primers and probes are listed in Table S2 in Supplementary Material). Nested PCR products were cloned into sequencing plasmid pCR4 (Invitrogen™) and sequenced using M13F and M13R primers as previously described (14). Sequence alignment was performed with MacVector with Assembler (version 12.5.0) and MEGA 6. Phylogenetic analyses were conducted in MEGA 6.

### Genotypic Assays

For sequences identical throughout the whole 1.2 kb, only one representative sequence was included in analysis. Truncated sequences with stop codons within the V3 region were excluded. Truncated sequences with stop codon outside the V3 region were included to maximize sample size, as these proviruses were functional at the time of entry. HIV-1 coreceptor usage was predicted using Geno2Pheno_[coreceptor]_ (G2P) (26), Web PSSM (both x4r5 and sinsi matrices) (27), and PhenoSeq-B (28).

### Phenotypic Assay for Coreceptor Tropism

Patient envelope sequences were cloned into expression plasmid pSVIII-Env by overlap extension PCR. In the first round PCR, the 1.2 kb V1–V5 fragment was amplified from the sequencing plasmid using primers E20F and CD4R as described above. The 5′gp120 fragment (upstream of the V1–V5 fragment) and 3′gp120 + gp41 fragment (downstream of the V1–V5 fragment) were amplified from plasmid HXB2.pAMP1 using primer sets Env-*Kpn*I (29) plus E20R (reverse and complementary to E20F) and CD4F (reverse and complementary to CD4R) plus Env-*Bam*HI (29). As a result, fragments 5′gp120 and V1–V5 overlapped by 18 bp at binding site of primer E20F/E20R and fragments V1–V5 overlapped by 24 bp at binding site of primer CD4F/CD4R (Figure S1 in Supplementary Material). In the second round overlap extension PCR, the above three fragments were mixed and amplified using primers Env-*Kpn*I and Env-*Bam*HI, and Expand high fidelity PCR system (Roche Diagnosis). PCR cycling consisted of an initial denaturation step at 94°C for 2 min, followed by 9 cycles of 94°C for 15 s, 60°C for 30 s, and 72°C for 2 min; then a further 20 cycles of 94°C for 15 s, 60°C for 30 s, and 72°C for 2 min but with a 5 s increase in extension time each cycle, followed by a final extension at 72°C for 7 min (30). The resulting fragment of approximately 2.1 kb, spanning the 5′*Kpn*I to 3′*Bam*HI restriction sites in HIV-1 *env*, was cloned into pSVIII-Env vector (Figure S1 in Supplementary Material).

Env-pseudotyped, luciferase reporter viruses were produced by transfection of 293 T cells with pCMVΔP1ΔenvpA, pHIV-1Luc (31), and pSVIIIpenv plasmids at a ratio of 1:3:1 using Lipofectamine 2000 (Invitrogen, Life Technologies). Supernatants were harvested 48 h later and clarified by filtration through 0.45 µm filters (32, 33).

The ability of Env-pseudotyped, luciferase reporter viruses to use CCR5 and/or CXCR4 was determined by single-round entry assays using the NP2 cells, which stably express CD4 together with CCR5 or CXCR4 as previously described (32). A cut-off was set to 5 × 10^4^ relative light unit based on the positive and negative controls.

### *In Vitro* Cell Culture and T Cell Receptor (TCR) Stimulation

Purified CD4^+^ T cell subsets were cultured in 96-well U-bottom tissue culture plates, at cell density of 2 to 2.5 × 10^6^/ml in 100 µl of RPMI-1640 GlutaMax™ medium (Gibco^®^, Life Technologies) supplemented with 10% of FCS (R10 medium), 100 U/ml of penicillin, 100 µg/ml of streptomycin (Gibco^®^, Life Technologies), and 0.5 µg/ml of purified mouse anti-human CD28 mAb (BD Pharmingen). TCR stimulation was achieved by coating the wells with 100 µl of 1 µg/ml purified mouse anti-human CD3 antibody at 4°C for a minimum of 2 h. Wells were rinsed twice with D-PBS before plating the cells. In some experiments, cytokines were added in cell culture as follows: recombinant human interleukin-6 at 20 ng/ml (R&D Systems) and rhIL-21 at 10 ng/ml (BIOSOURCE, CA, USA).

### Fusion Assay for Measurement of Viral Entry Efficiency

Plasmids encoding HIV-1 provirus used were pNL4.3 and pAD8-NL4.3. pNL4.3 was obtained through the NIH AIDS Research Reagent Program (Cat #: 114). pAD8-NL4.3 was constructed by replacing the *env* region of pNL4.3 with *env* from HIV-1 AD8. Plasmid pNL4.3 or pAD8-NL4.3, and the plasmid encoding a β-lactamase-Vpr (pBlaM-Vpr) chimera (kind gift of Dr. Marielle Cavrois). pBlaM-vpr were cotransfected into 293 T cells to make CXCR4- or CCR5-using HIV-BlaM as previously described (34).

Human tonsillar mononuclear cells were resuspended in R10 medium at 10^7^/ml. Then, 100 µl of cell suspension was loaded into each well of the 96-well V-bottom tissue culture plate. Maraviroc or AMD3100 were added to the designated wells at a working concentration of 20 nM. Cells were incubated with drug at 37°C for 10 min. HIV-BlaM was added to the designated wells at TCID50 = 10^6^/ml and cells were spin inoculated at 800 × *g* for 1 h at 18°C. Following spin inoculation, cells were resuspended in 100 μl/well of fresh R10 medium and incubated at 37°C with 5% CO_2_ for 2 h. Following incubation, cells were centrifuged at 500 × *g* for 2 min, resuspended in 100 μl/well of CCF2-AM loading solution (from LiveBLAzer™ FRET—B/G Loading Kit, Invitrogen Life Technologies) and incubated with loading solution at room temperature in the dark for 1 h. Following loading, cells were centrifuged at 500 × *g* for 2 min and washed with 200 μl/well of development solution (CO_2_-independent medium supplemented with 10% FCS and 2.5 mM probenecid). Cells were then resuspended in 200 μl/well of development solution and incubated at room temperature in the dark for 16 h.

### Statistical Analysis

Statistical analysis was performed using GraphPad Prism^®^, Version 5 or 6 (GraphPad Software, Inc., La Jolla, CA, USA). Unless otherwise indicated, data represent the median ± interquartile (IQR), and non-parametric analyses were performed with *p* < 0.05 considered statistically significant.

## Results

### Human Tfh Cells Carry Proviral Envelope Sequences That Are Indistinguishable from Other Memory Cells and Are CCR5 Tropic

We previously obtained cryopreserved single-cell suspensions of splenic tissue from five patients with chronic untreated HIV infection (23), from which CD3^+^CD4^+^CD19^−^ cells were sorted into naïve CD45RA^+^ICOS^−^ and resting memory cell (CD45RA^−^ICOS^−^), and two subsets of Tfh defined as (i) CXCR5^int+^ Tfh (CD45RA^−^ICOS^+^PD-1^hi+^CXCR5^int+^) and (ii) CXCR5^hi+^ GC Tfh (CD45RA^−^ICOS^+^PD-1^hi+^CXCR5^hi+^), as shown in Figure S2 in Supplementary Material. We found that both subsets of Tfh cells contained increased levels of proviral DNA (23), as shown in Figure S3 in Supplementary Material.

The HIV-1 proviral V1–V5 regions of gp120 obtained from the different subsets were sequenced from three subjects and subjected to phylogenetic analysis. As expected, sequences segregated by individual subject (Figure S4 in Supplementary Material).

**Figure 1:**
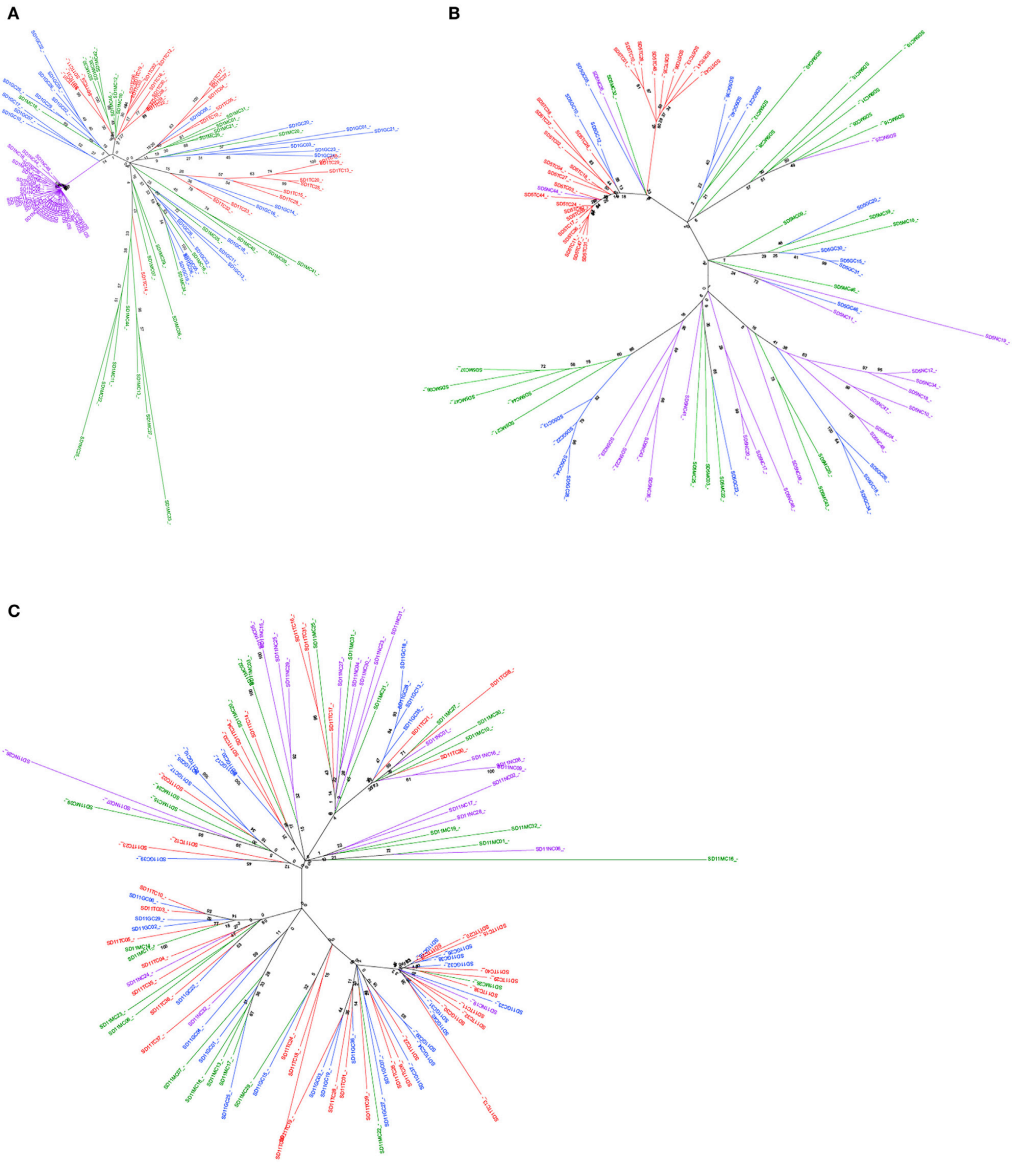

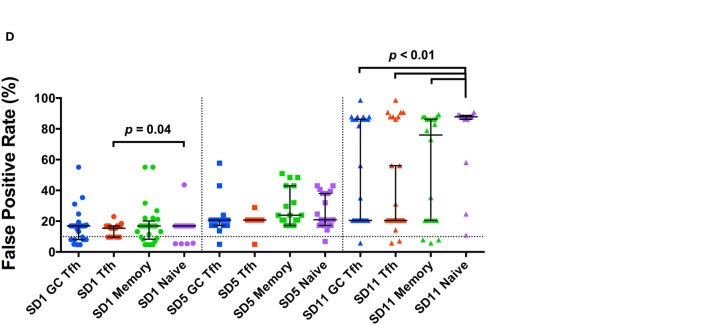
**Phylogenetic analysis of HIV-1 gp120 proviral DNA clonal sequences**. **(A–C)** Phylogenetic trees constructed using envelope clones derived from four CD4^+^ T cell subsets from each patient **(A)** SD1, **(B)** SD5, and **(C)** SD11. Branches and clone names are color coded for different cell subsets [blue: GC T follicular helper (Tfh); red: Tfh; green: memory; purple: naïve]. Sample names consist of subset name and clone number. Bootstrapped consensus trees, inferred from 1,000 replicates, were constructed using the neighbor-joining method with the bootstrap values shown at the branches. **(D)** Geno2pheno false positive rate (FPR) values of *env* clones in different subsets. Data points of different subsets are distinguished by color and data points of different patients are distinguished by symbol shape (SD1: circle; SD5: square; SD11: triangle). FPR of 10% is shown by dotted line. Medians and interquartile ranges are shown unless otherwise stated.

However, within each individual subject, the sequences from Tfh and GC Tfh were in general intermingled with sequences from the other CD4^+^ T cell subsets (Figures 1A–C). In patient SD1, there was a distinct cluster arising from the naive cell population, but the sequences from Tfh, GC Tfh, and memory cell populations interspersed, suggesting infection of naïve CD4^+^ T cells by a distinct quasi-species (Figure 1A). For patient SD5, sequences from Tfh cells tended to cluster, however, within these sequences from the other three CD4^+^ T cell subsets were mixed. The majority of sequences from GC Tfh and memory and naïve CD4^+^ T cells were intermixed (Figure 1B). For patient SD11, sequences from each subset were intermingled with sequences from other subsets (Figure 1C). The lack of distinct envelope sequence clusters arising from viruses infecting Tfh populations compared to non-Tfh memory cells, suggests that the viral envelopes used to infected Tfh were similar to those used to infect other memory cells.

The sequences were submitted to several tropism prediction algorithms. Using Geno2pheno_[coreceptor]_ (G2P), the false positive rate (FPR) values were compared and no significant difference was observed between any two memory CD4^+^ T cell subsets within the same patient (Figure 1D). In SD1 and SD11, the V3 loop sequences from the naïve CD4^+^ T cell subsets had significantly higher FPRs than sequences from one or more memory CD4^+^ T cell subsets within the same individual, suggesting that these naïve cells were also infected with typical CCR5-using variants.

Similarly, analysis of the V3 loop sequences by either PSSMx4r5 or PSSMsinsi algorithms indicated the sequences were typical CCR5-using (Figure S5 in Supplementary Material) (27). Only a small minority of sequences were predicted to be as CXCR4-using by PhenoSeq-B (28). These results were highly concordant with those predicted by G2P using an FPR cut-off of 10% (Table S3 in Supplementary Material).

The phenotypic tropism of envelope clones was studied in 10 functional envelope clones derived from the available DNA. All were CCR5-using, including six from GC Tfh and Tfh. None were found to be CXCR4-using (Figure S6 in Supplementary Material).

Taken together, these results on viral sequences derived from human Tfh are consistent with our previously reported findings in macaques (14). In both studies, the viral envelopes derived from Tfh were predominantly CCR5-using and had similar sequences and tropism to those found in other memory cells. Thus, direct infection of Tfh, which are predominantly CCR5 negative, by non-CCR5 utilizing HIV strains does not explain the high levels of infection observed.

### *Bcl6* mRNA and Cell Surface CCR5 Expression in Macaque PD-1^int+^ Precursors of Tfh Cells

Therefore, we explored the mechanism by which a CCR5-using virus infects a predominantly CCR5-negative population. We used the SIV infection model and macaque cells for functional assays, since substantial numbers of LN and spleen cells could be obtained from both SIV-infected and -uninfected pigtail macaques through excision biopsies (Table S1 in Supplementary Material). We have previously shown that macaque Tfh are normally CCR5 negative (14), consistent with the literature (5), although following SIV infection there may be a small proportion of Tfh that express low levels of CCR5 (14). Therefore, we hypothesized that macaque Tfh were infected at a putative CCR5^+^ precursor stage, and that SIV infection was maintained during their differentiation into Tfh.

**Figure 2:**
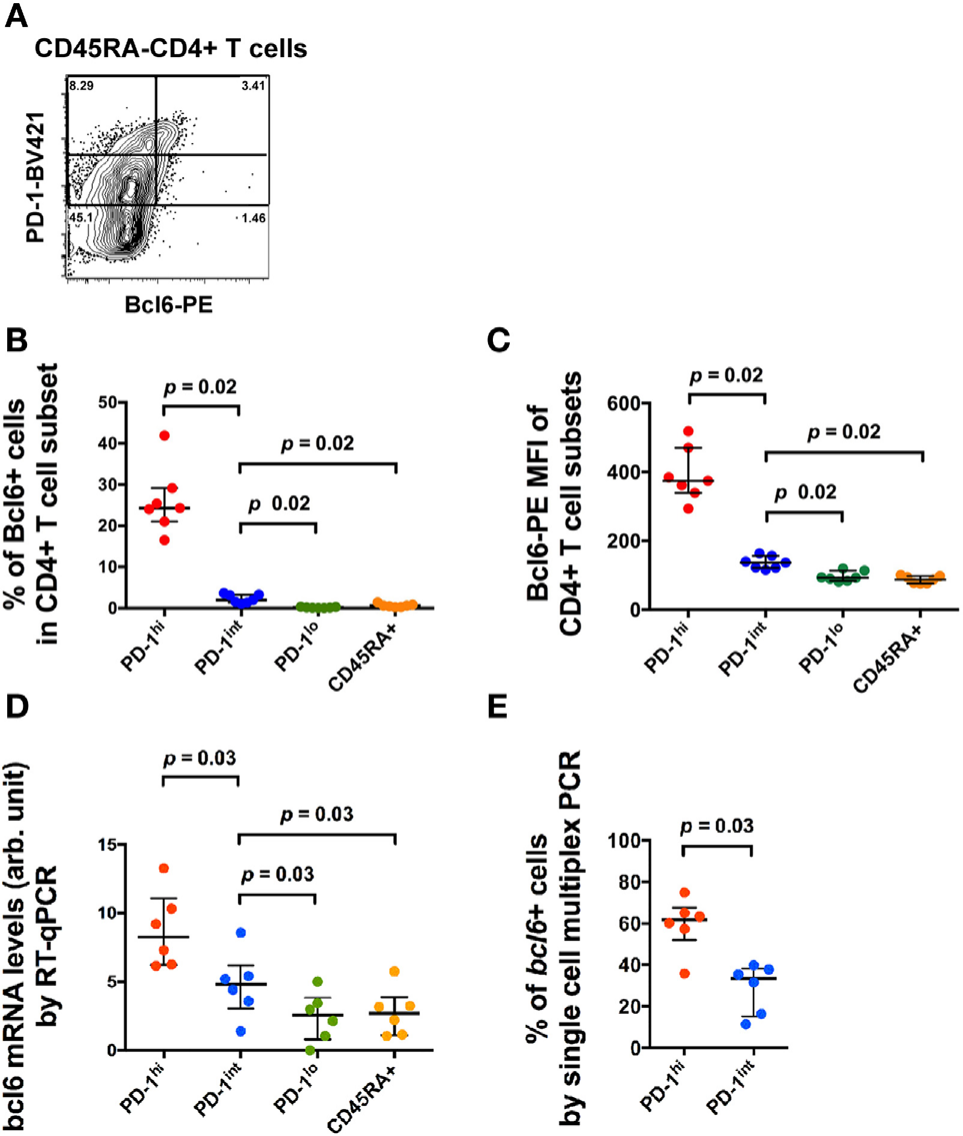
**Bcl6 expression in different CD4^+^ T cell subsets**. **(A)** Representative flow histogram showing Bcl6 expression in subsets of CD45RA^−^ memory CD4^+^ T cells defined by different expression levels of PD-1. Expression of Bcl6 in B cells was used to set the analytical gates. **(B)** Summary data of the proportion of Bcl6^+^ cells within different CD4^+^ T cell subsets (blue: CD45RA^−^PD-1^hi+^, red: CD45RA^−^PD-1^int+^, green: CD45RA^−^PD-1^lo+^, orange: CD45RA^+^). **(C)** Summary data of the MFI of Bcl6 in these CD4^+^ T cell subsets. **(D)** *bcl6* mRNA expression in bulk sorted CD4^+^ T cell subsets by conventional RT-qPCR assay. **(E)** Proportion of cells expressing *bcl6* mRNA in single-cell sorted CD4^+^CD45RA^−^PD-1^hi+^ and PD-1^int+^ subsets of six macaques (three SIV-uninfected macaques and three SIV-infected macaques) (see Table S2 in Supplementary Material). Wilcoxon test was used to determine significance unless otherwise stated.

T follicular helper can be identified within CD4^+^CD45RA^−^ T cells within single-cell suspensions of macaque LN cells by the coexpression of high levels of PD-1 (PD-1^hi+^) and Bcl6^+^ (14). As we and others have previously reported, the numbers of these cells were increased in SIV-infected macaques (14, 15, 35). A small proportion of memory CD4^+^ T cells that expressed intermediate levels of PD-1 (PD-1^int+^) also exhibited low level expression of Bcl6 by flow cytometry (Figures 2A–C). These cells were of unclear significance so we studied their phenotype in more detail. The increased, but low level expression of Bcl6 in PD-1^int+^ memory CD4^+^ T cells was confirmed by RT-PCR analysis of *bcl6* mRNA from purified PD-1^int+^ cells (Figure 2D). Comparison of the PD-1^hi+^ and PD-1^int+^ memory CD4^+^ T cell populations for *bcl6* mRNA expression by single-cell analysis, showed that 60% (median) of the former and 35% of the latter were *bcl6*^+^ (Figure 2E).

**Figure 3:**
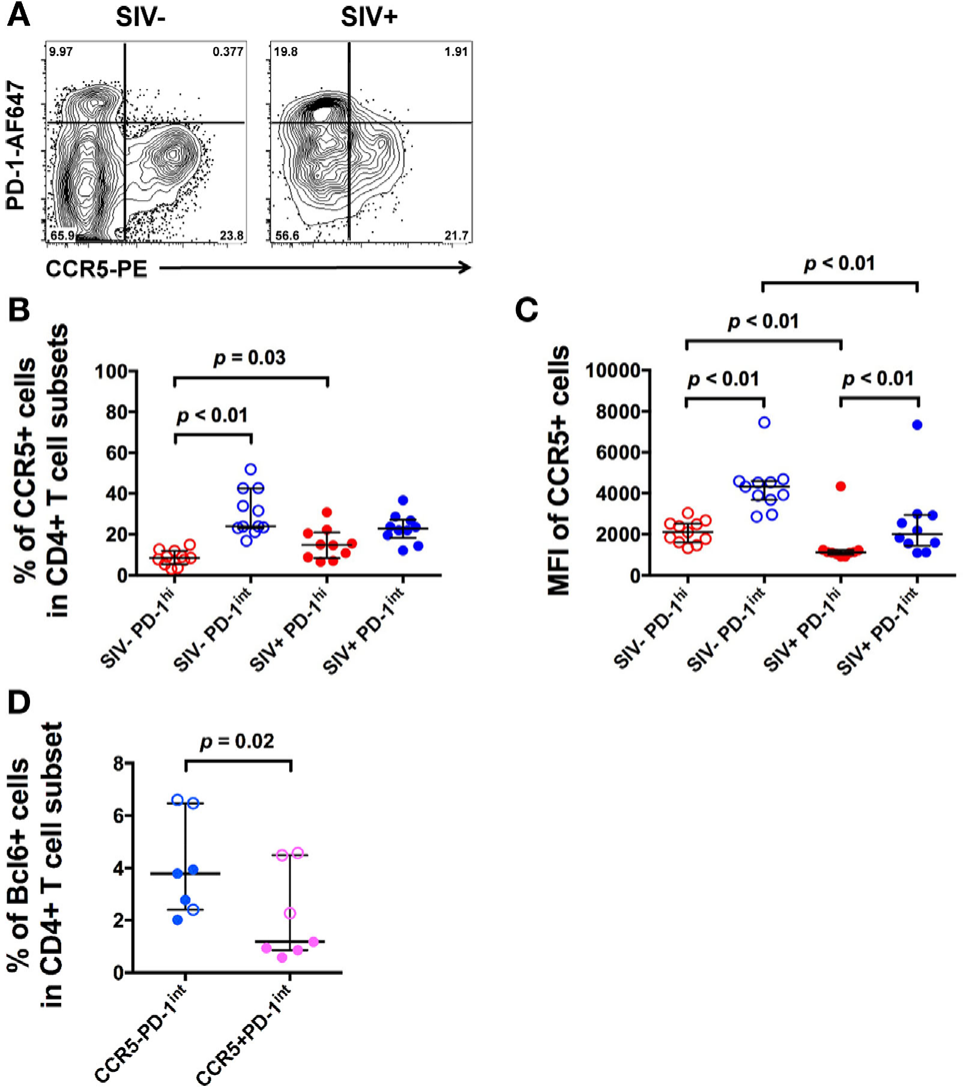
**CCR5 expression varies with PD-1 expression in CD4^+^ T cell subsets in uninfected and SIV-infected macaques**. **(A)** Representative flow plots showing CCR5 expression on CD4^+^ T cell subsets in uninfected and SIV-infected macaques. Cells are gated on CD45RA^−^ memory CD4^+^ T cells. **(B)** Frequencies of CCR5^+^ cells in PD-1^hi+^ (red) and PD-1^int+^ (blue) subsets in uninfected (open symbols) and SIV-infected macaques (closed symbols). **(C)** CCR5 MFI in the same PD-1^hi+^ and PD-1^int+^ subsets. Wilcoxon test was used to determinate significance between two subsets within the same group (SIV^−^ or SIV^+^ group). **(D)** Summarized data of the frequencies of Bcl6^+^ cells in *ex vivo* CCR5^−^PD-1^int^ and CCR5^+^PD-1^int^ cells in SIV-uninfected (open symbols) and SIV-infected (closed symbols) macaques. Mann–Whitney test was used to determine significance between two groups of the same subset.

The *bcl6*^+^ subset of the PD-1^int+^ population may represent Tfh precursors. Therefore, we then measured CCR5 cell surface expression in the same PD-1^hi+^ and PD-1^int+^ subsets from macaque LNs, using an optimized indirect immunofluorescence staining method in order to maximize the CCR5 signal-to-noise ratio (14). The overwhelming majority of CCR5^+^ cells were in the PD-1^int+^ subset of memory CD4^+^ T cells (Figure 3A).

In pigtail macaques with untreated SIV infection, there was a small, but significant increase in the number of CCR5^+^PD-1^hi+^ cells compared to healthy SIV-uninfected macaques (*p* = 0.03, Figure 3B). However, the extent of expression of CCR5, as judged by MFI was significantly lower in the PD-1^hi+^ population than in the PD-1^int+^ cell subset, whether or not macaques was infected (*p* < 0.01, Figure 3C).

Bcl6 protein expression in *ex vivo* CCR5^–^PD-1^int^ cells was significantly higher than in *ex vivo* CCR5^+^PD-1^int^ cells (*p* = 0.02, Figure 3D), suggesting that majority of the pre-Tfh cells were CCR5^−^ but a small proportion was CCR5^+^.

### *In Vivo* Infection of CCR5^+^PD-1^int+^ and CCR5^−^PD-1^hi+^ Memory CD4^+^ T Cells

**Figure 4:**
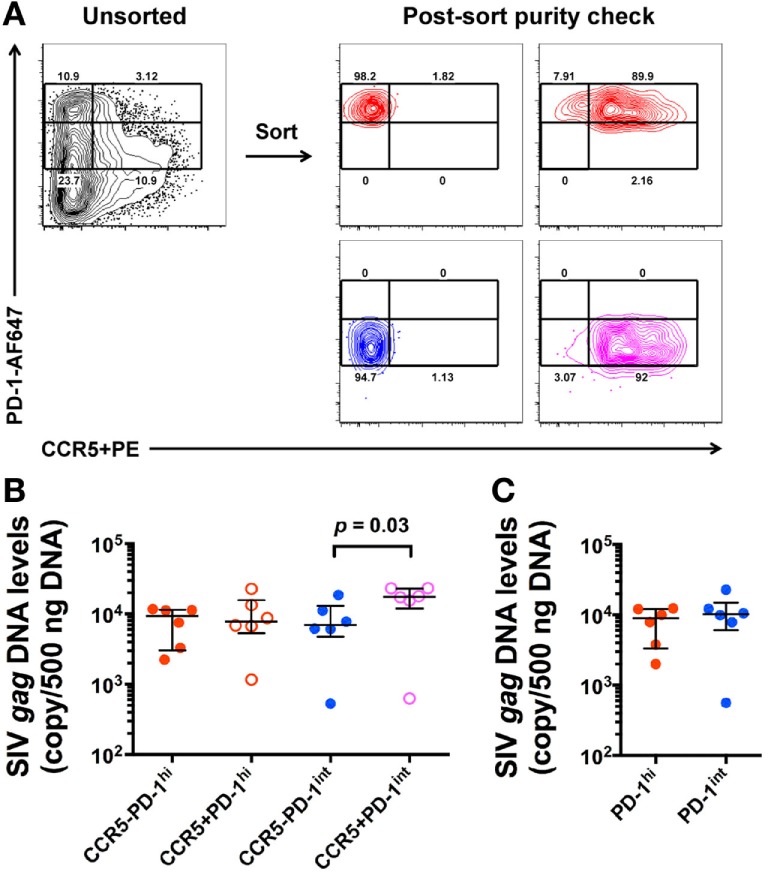
**SIV-*gag* DNA levels in *ex vivo* CCR5^+^ and CCR5^−^ CD45RA^−^ memory CD4^+^ T cells**. **(A)** Representative flow plots showing gating strategies for sorting of *ex vivo* CD4^+^ T cell subsets from macaque lymph node mononuclear cells (left panel) and purity check post sorting (right panels). CD45RA^−^ memory CD4^+^ T cells were examined against PD-1 and CCR5 expression. **(B)** Normalized SIV-*gag* DNA levels (copies per 500 ng DNA) in CCR5^−^PD-1^hi^ (red closed), CCR5^+^PD-1^hi+^ (red open), CCR5^−^PD-1^int+^ (blue closed), and CCR5^+^PD-1^int+^ (magenta open) subsets (*n* = 6). **(C)** Relative contribution of PD-1^hi+^ (red) and PD-1^int+^ (blue) CD4^+^CD45RA^−^ T cells to SIV-*gag* DNA levels after adjustment for cells numbers in each of these subsets.

Since we found CCR5^+^PD-1^hi+^ Tfh cells in SIV^+^ macaques, we investigated the extent to which these contributed to the high levels of proviral DNA levels in Tfh. LN mononuclear cells were stained for CCR5 with the indirect immunofluorescence method and CD45RA^−^ memory CD4^+^ T cells were sorted based on PD-1 and CCR5 expression (Figure 4A). SIV-*gag* DNA levels in the CCR5^+^PD-1^int+^ subset were significantly higher than those found in the CCR5^−^PD-1^int+^ subset (*p* = 0.03), whereas within PD-1^hi+^ cells, the SIV-*gag* DNA levels were not higher in the CCR5^+^PD-1^hi+^ compared to the CCR5^−^PD-1^hi+^ subsets (Figure 4B). Overall, CCR5^−^PD-1^hi+^, CCR5^−^PD-1^int+^ cells, and CCR5^+^PD-1^hi+^ cells had similar levels of SIV-*gag* DNA on a per cell basis (Figure 4B). Regardless of CCR5 carriage PD-1^int+^ and PD-1^hi+^ cells have similar levels of SIV-*gag* on a per cell basis (Figure 4C), consistent with previous observations (14).

### *In Vitro* Activation of PD-1^int+^ Memory CD4^+^ T Cells Leads to Upregulation of PD-1 and Bcl6, Including SIV-Infected Pre-Tfh

**Figure 5:**
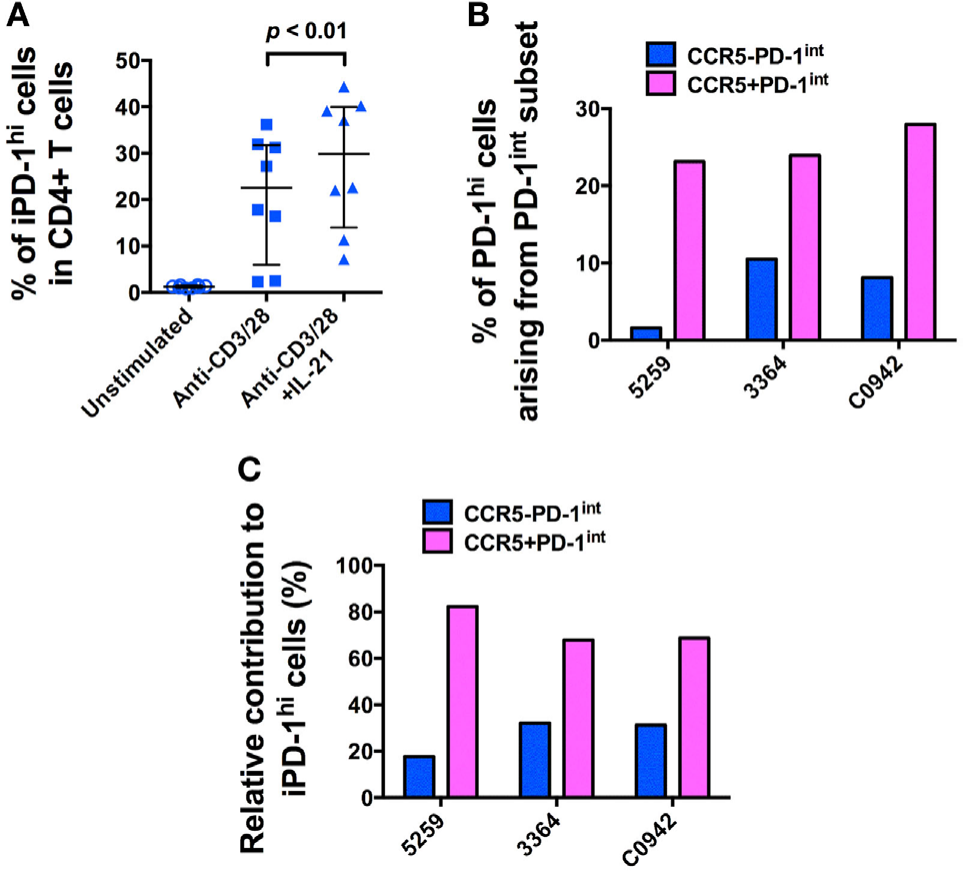
**The majority of *in vitro*-induced PD-1^hi+^ (iPD-1^hi+^) cells arise from CCR5^+^PD-1^int+^ cells**. **(A)** Significant increase in the proportion of cells expressing PD-1^hi+^ arising from isolated CD4^+^CD45RA^−^PD-1^int+^ T cells stimulated with anti-CD3/CD28 with or without IL-21 for 72 h. **(B)** Bar chart showing proportion of iPD-1^hi+^ cells arising from stimulated PD-1^int+^ subsets in three separate uninfected macaques. *X*-axis shows animal ID. **(C)** Bar chart showing the relative contribution of CCR5^−^ (blue) and CCR5^+^ (magenta) PD-1^int+^ cells to iPD-1^hi+^ cells, after adjustment for their population size in *ex vivo* samples.

Since similar levels of SIV DNA were found in both the CCR5^−^PD-1^hi^ and the CCR5^+^PD-1^hi^ subsets of Tfh, we hypothesized that the majority of infected Tfh had become infected before differentiating into mature Tfh. We determined whether we could induce differentiation of Tfh, from precursor Tfh contained within purified PD-1^int+^CD45RA^−^CD4^+^ T cells, by activation *via* the TCR, in the presence or absence of IL-21. Over 20% of purified PD-1^int+^ memory CD4^+^ T cells became PD-1^hi+^ after incubation for 72 h with anti-CD3, anti-CD28 monoclonal antibodies (mAb), and addition of IL-21 significantly augmented this effect (*p* < 0.01, Figure 5A; Figure S7A in Supplementary Material). Importantly, these induced PD-1^hi+^ (iPD-1^hi+^) cells arose from both the CCR5^−^PD-1^int+^ and CCR5^+^PD-1^int+^ populations, with 18–32% arising from CCR5^−^PD-1^int+^ cells, and the remainder arising from the CCR5^+^ cells (Figures 5B,C; Figure S7B in Supplementary Material).

**Figure 6:**
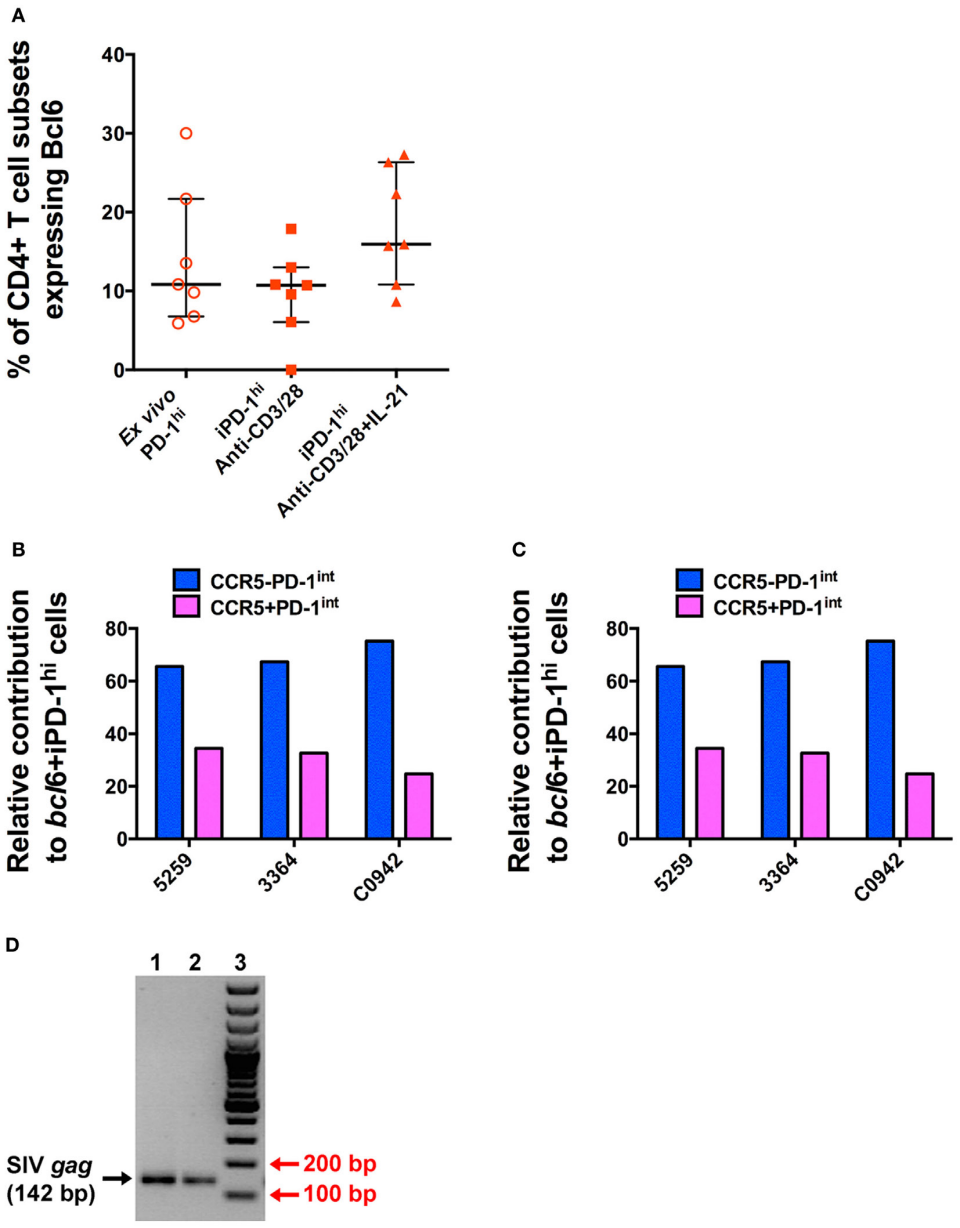
**T cell receptor stimulation of purified CD4^+^CD45RA^−^PD-1^int+^ T cells induces Bcl6 expression to levels similar to that seen in T follicular helper (Tfh) isolated *ex vivo* and allows SIV-*gag* to be isolated from induced Tfh**. **(A)** The proportion of *ex vivo* and induced PD-1^hi+^ (iPD-1^hi+^) cells that express Bcl6 is similar. **(B)** Relative proportions of iPD-1^hi+^ cells expressing *bcl6* mRNA after adjustment for the population size of *ex vivo* CCR5^−^ (blue) and CCR5^+^ (magenta) PD-1^int+^ cells. **(C)** Relative proportion of iPD-1^hi+^ cells expressing Bcl6 protein by flow cytometry after adjustment for the population size of *ex vivo* CCR5^−^ and CCR5^+^PD-1^int+^ cells. **(D)** SIV-*gag* is present in iPD-1^hi+^ cells from SIV-infected macaques. Image of 2% agarose gel showing correct size band of SIV-*gag* (142 bp, indicated by black arrow, lane 1 and 2, in replicate) after nested PCR. Red arrow indicates the 200 and 100 bp bands of the DNA ladder (lane 3). The image is representative of three independent experiments.

In these iPD-1^hi+^ cells, the expression of Bcl6 protein was also upregulated, to levels similar to those found in *ex vivo* PD-1^hi+^ Tfh populations (Figure 6A). After adjusting for the original population size of *ex vivo* CCR5^−^ and CCR5^+^ cells within the total PD-1^int+^ cells, the number of iPD-1^hi+^ cells expressing *bcl6* mRNA or protein, arising from CCR5^−^PD-1^int+^ subset was more than two times greater than those arising from the CCR5^+^PD-1^int+^ cells (Figures 6B,C). Altogether, these results suggest that a majority of iPD-1^hi+^ cells were derived from CCR5^+^PD-1^int+^ cells, while the CCR5^−^PD-1^int+^ cells have relatively more Bcl6^+^ Tfh precursor cells.

We next demonstrated that infected PD-1^int+^ cells can carry proviral DNA while differentiating into iPD-1^hi+^ cells following TCR engagement. Then, 48 h after stimulation of purified PD-1^int+^ cells from SIV-infected macaque LN samples, the iPD-1^hi+^ were sorted, and SIV-*gag* DNA detected by nested PCR. Then, 500–2,000 iPD-1^hi+^ cells were recovered in three independent experiments. SIV-*gag* DNA was detected in each sample (Figure 6D). The results suggest that differentiation of SIV DNA-infected CCR5^+^PD-1^int^ to a Tfh phenotype is a plausible pathway for a CCR5-tropic virus to be found within a predominately CCR5-negative cell population such as Tfh.

### Human PD-1^hi+^ Tfh Cells Have a Relatively High Proportion of CCR5-Expressing Cells

However, we also found a second, possibly more direct pathway for HIV-1/SIV infection of Tfh. Despite being thought to be CCR5 negative, there was a small population of PD-1^hi+^ cells in SIV-infected macaques that were CCR5^lo+^, detected by our indirect immunofluorescence staining method.

**Figure 7:**
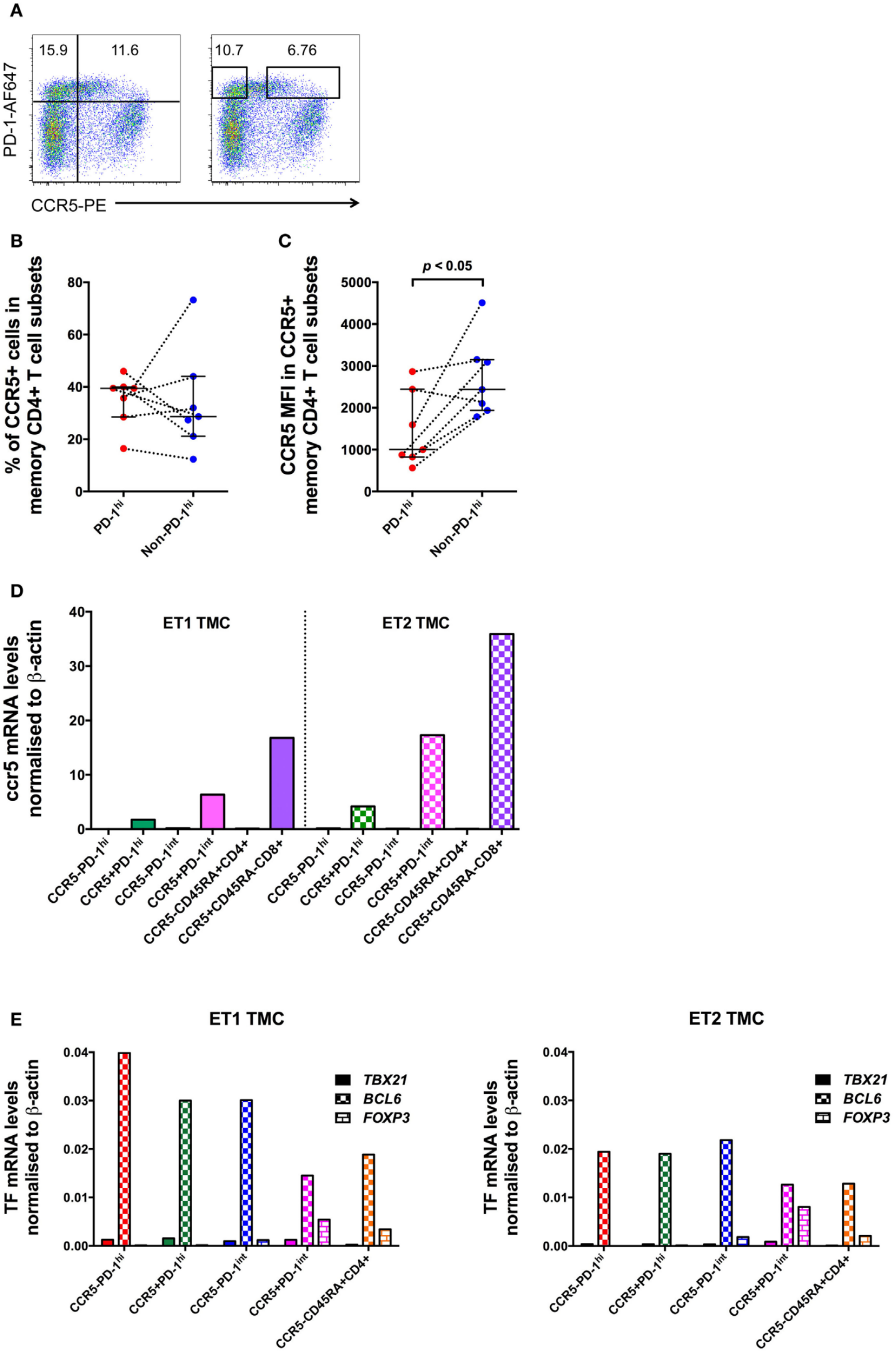
**CCR5 and Bcl6 expression by human tonsillar T follicular helper (Tfh) from HIV-uninfected subjects**. **(A)** CCR5 gating strategies for analysis and for sorting of human tonsillar Tfh from single-cell suspensions. CD45RA^−^CD4^+^ T cells are plotted against PD-1 vs. CCR5 and divided into four subsets based on PD-1 and CCR5 expression (left panel) for analysis. The boxes in the right panel show gating strategy for sorting CCR5^−^PD-1^hi^ and CCR5^+^PD-1^hi^ cells with conserved box gates to ensure the sorted subsets are of high purity. CCR5 protein expression on these cells as determined by panel **(B)** proportion of cells positive for CCR5 and **(C)** MFI of CCR5^+^ memory CD4^+^ T cells subsets. **(D)** ccr5 mRNA levels of purified memory CD4^+^ T cells subsets. **(E)** RT-qPCR of mRNA from human tonsillar memory CD4^+^ T cell subsets demonstrates that the predominant transcription factor in PD-1^hi+^ cells is *Bcl6*. Mann–Whitney test was used to determinate significance.

Surprisingly, examination of mononuclear cell suspensions from HIV seronegative patients having tonsillar excision biopsies showed that a substantial proportion of PD-1^hi^ Tfh cells expressed CCR5 (Figure 7A). To confirm this, lymphoid tissue samples from seven healthy uninfected controls were sourced for the measurement of CCR5 expression on PD-1^hi+^ Tfh (Table S4 in Supplementary Material). Using the 2D7 antibody clone to stain for CCR5, a median 39.5% (IQR: 28.5–40.0%) of the PD-1^hi^ subset of CD4^+^ memory cells was CCR5^+^. There was no significant difference in the proportion of CCR5^+^ cells between PD-1^hi+^ and non-PD-1^hi+^ cells (median 28.7%; IQR 21.2–44.1%) (Figure 7B). However, the CCR5 MFI in CCR5^+^PD-1^hi+^ cells was significantly lower than that in CCR5^+^non-PD-1^hi+^ cells (*p* < 0.05, Figure 7C).

Tonsillar mononuclear cells were sorted into CCR5^−^PD-1^hi+^, CCR5^+^PD-1^hi+^, CCR5^−^PD-1^int+^ and CCR5^+^PD-1^int+^ subsets of CD45RA^−^ memory CD4^+^ T cells, as well as CCR5^−^CD45RA^+^ naïve CD4^+^ T cells, and CCR5^+^CD45RA^−^ memory CD8^+^ T cells. The purity of the sorted cells was between 94.2 and 100%. *ccr5* mRNA was detected at high levels in all CCR5^+^ subsets but was absent in all CCR5^−^ subsets (Figure 7D), strongly suggesting that the CCR5 expression is a true property of at least a subset of Tfh.

Furthermore, cell sorted CCR5^−^CD45RA^−^PD-1^hi+^ and CCR5^+^CD45RA^−^PD-1^hi+^ subsets of tonsillar CD4 T cells both exhibited equivalent very high expression of *bcl6* mRNA (Figure 7E) without substantial expression of T-bet or FoxP3 suggesting that human PD-1^hi^ CD4^+^ memory T cells isolated from lymphoid tissue are almost exclusively Tfh cells, regardless of their surface CCR5 expression.

### Infection of Human Pre-Tfh/Tfh Is Mediated through CCR5

The results of the preceding studies suggest that in pigtail macaques PD-1^hi+^ Tfh cells are infected either as CCR5^+^PD-1^int+^ pre-Tfh cells, which then differentiate and upregulate PD-1, or as CCR5^low+^PD-1^hi+^ cells, the proportion of which increases during SIV infection. These mechanisms could also apply in humans.

A quantitative virus-cell fusion assay was used to monitor the propensity of *in vitro* infection of these primary cell subsets with viruses expressing envelopes of known tropism, AD8 for CCR5-using HIV-1, and NL4.3 for CXCR4-using HIV-1, respectively. This assay allows combined detection of virus-cell fusion and cell surface phenotype 18 h after virus inoculation to analysis, essentially a single round of infection (34).

**Figure 8:**
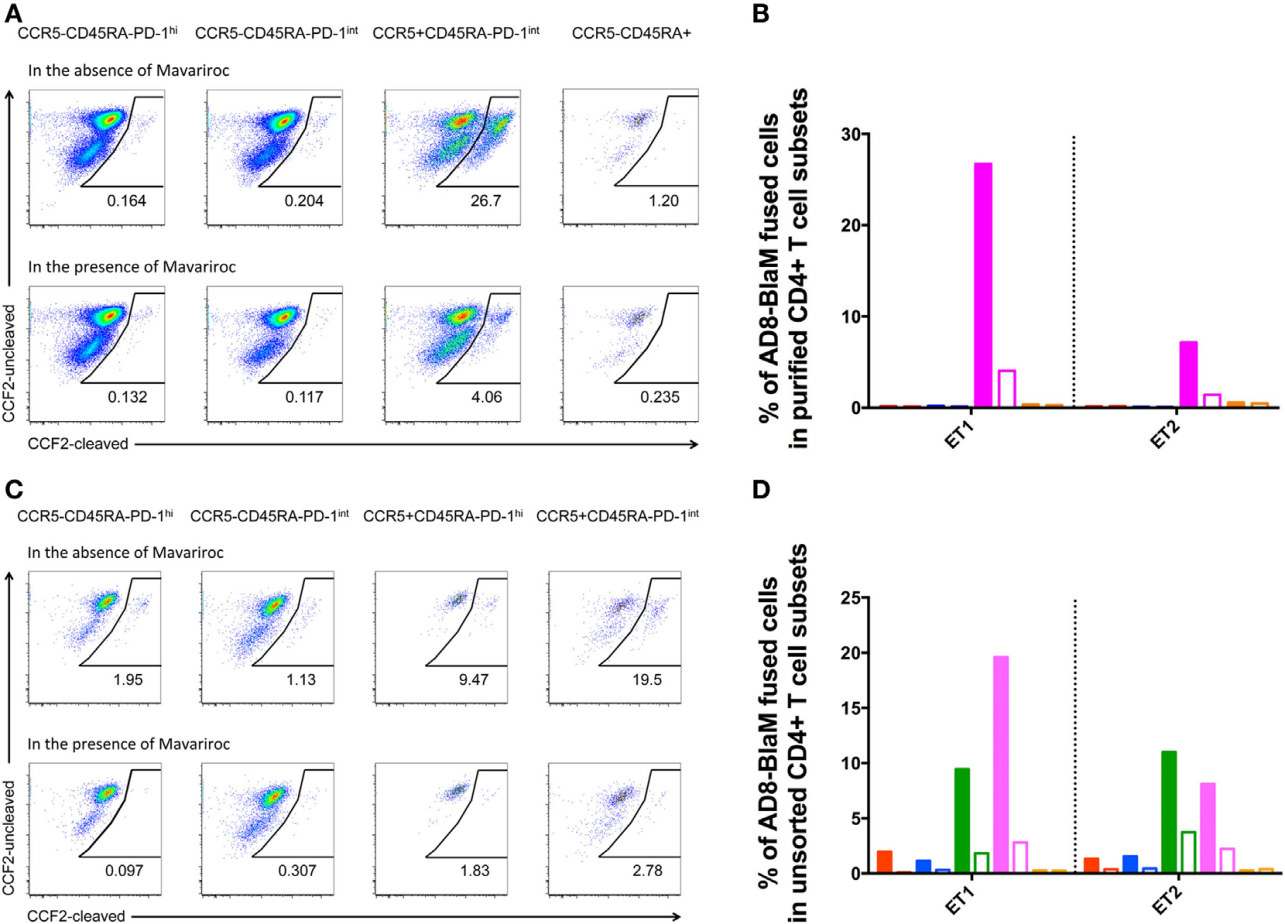
**CCR5-tropic virus fuses with human pre-Tfh isolated from human tonsils**. **(A,C)** Representative flow plots showing fusion of AD8-BlaM with **(A)** sorted-purified CD4^+^ T cell subsets and **(C)** unsorted, human tonsillar mononuclear cells. In each of panels **(A,C)**, the top row of flow plots show virus fusion in the absence of entry inhibitors. Bottom row of flow plots shows virus fusion in the presence of Maraviroc. The phenotype and % of fused cells are shown for each subset. **(B,D)** Percentage of AD8-BlaM fused cells in panel **(B)** purified CD4^+^ T cell subsets and in panel **(D)** CD4^+^ T cell subsets from unsorted samples (red: CCR5^−^CD45RA^−^PD-1^hi+^, blue: CCR5^−^CD45RA^−^PD-1^int+^, magenta: CCR5^+^CD45RA^−^PD-1^int+^, orange: CCR5^−^CD45RA^+^), in the absence (filled) and presence (clear) of Maraviroc.

There was fusion of CCR5-using AD8-BlaM with purified CCR5^+^CD45RA^−^PD-1^int^ cells, in two independent experiments, which was efficiently inhibited by the CCR5^−^ inhibitor, Maraviroc (fusion reduced to 3.5 and 1.0%, respectively) (Figures 8A,B). By contrast, there was no fusion of AD8-BlaM into purified CCR5^−^CD45RA^−^PD-1^hi+^, CCR5^−^CD45RA^−^PD-1^int+^, and CCR5^−^CD45RA^+^CD4^+^ T cell subsets (Figures 8A,B).

Fusion experiments were also performed on unsorted cells allowing characterization of the CCR5^+^PD-1^hi+^ subpopulation that was too small to allow collection of enough cells to allow examination as an isolated purified population. These experiments recapitulated the findings in the sorted populations showing efficient fusion with the CCR5^+^CD45RA^−^PD-1^int+^ population but also revealed fusion with the small but clearly present CCR5^+^CD45RA^−^PD-1^hi+^ population (Figures 8C,D).

CXCR4-using NL4.3-BlaM was included in the assays as a positive control. Fusion of NL4.3-BlaM was observed for all purified subsets, and CD4^+^ T cell subsets in unsorted samples (Figure S8 in Supplementary Material). AMD3100 resulted in complete blocking of this fusion (Figure S8 in Supplementary Material).

## Discussion

T folliicular helper cells characteristically express the chemokine receptor CXCR5 that directs pre-Tfh, and subsequently Tfh, to B cell areas of organized lymphoid tissue, where they play a critical role in helping GC B cells to produce high-affinity, class-switched antibodies and to develop B cell memory. Conversely, Tfh reportedly lack expression of CCR5 in macaques, allowing them to maintain their location in the GC and not respond to inflammatory chemokines (7, 14). Despite this lack of CCR5 coreceptor expression, we and others had previously found that they carry a similar or higher SIV burden compared to other subsets of CD4^+^ memory T cells in infected macaques (14, 15, 36), and these viruses had proviral sequences that encoded CCR5-tropic envelopes that were not distinguishable from the *env* sequences found in other CD4^+^ T cell memory subsets (14). We had also previously shown that human Tfh are infected to at least the same degree as other CD4^+^ memory T cells (23).

By analyzing the gp120 sequences with three different genotypic coreceptor usage prediction algorithms and by a phenotypic assay, we provide the first evidence that proviruses from human splenic CD4^+^ T cells of HIV-infected patients have CCR5-using envelopes, and the envelope sequences found in Tfh subsets and non-Tfh memory CD4^+^ T cells are in general intermingled, suggesting a similar mechanism of entry across each of these CD4^+^ memory T cell subsets. These findings recapitulate our previous observations made in SIV infection of pigtail macaque Tfh cells (14). The challenge was then to explain how this apparent preferential infection occurred in a population of cells with minimal expression of CCR5.

By studying LNs from both healthy and SIV-infected pigtail macaques, we found an abundant PD-1^int+^ memory CD4^+^ T cells that displayed a pre-Tfh phenotype, characterized by expression of Bcl6, the master TF for Tfh cells. A substantial subset of these cells expressed CCR5. Further, upon activation through the TCR, these cells upregulated both PD-1 and Bcl6, consistent with development of a Tfh phenotype. Others, using similar stimulation protocols, have independently demonstrated the induction of Tfh from these precursor cells (36).

We confirmed that the PD-1^int+^ cells are heterogeneous with respect to CCR5 expression, as we had originally shown (14), but extended this to show that, although the CCR5^+^ subset was a significant source of the iPD-1^hi+^ cells, the CCR5^−^PD-1^int+^ subset was more highly enriched for Tfh cell precursors as judged by expression of Bcl6. These results suggest that the majority of Tfh cells, even as precursors (at the stage of PD-1^int+^), do not express CCR5. However, this minority population of CCR5^+^ Tfh cell precursors appears to be the main source of the pool of SIV-infected Tfh cells. The *in vitro* activation by TCR activation of the CCR5^+^ subset of PD-1^int+^ cells isolated from the LNs of SIV-infected macaques, reproducibly resulted in iPD-1^hi+^ Bcl6^+^ cells that contained SIV-*gag* DNA. These results robustly suggest that infected PD-1^int+^ cells can carry proviral DNA and further differentiate into PD-1^hi+^ cells after stimulation through the TCR, implying a pathway for the production of SIV or HIV DNA-infected Tfh cells *via* earlier infection of CCR5^+^ pre-Tfh cells.

The role of CCR5 expression in infection of the PD-1^int+^ pre-Tfh cells was demonstrated using a quantitative, flow-cytometric virus-cell fusion assay (34), which enabled the simultaneous detection of fusion of HIV-1 and cell surface markers. CCR5^−^ pre-Tfh and other CCR5^−^ CD4^+^ T cells from human tonsils were resistant to fusion of CCR5-using HIV-1, but were permissive to fusion of CXCR4-using HIV-1. By contrast, CCR5^+^ pre-Tfh were permissive to CCR5-using virus and this process could be specifically inhibited by the CCR5 antagonist, Maraviroc. These results suggest that it is unlikely that the CCR5^−^ Tfh cells can be directly infected with CCR5-using HIV-1, supporting the conclusion derived from the macaque studies that infection of Tfh was either a result of differentiation of infected pre-Tfh cells, or through transient upregulation of CCR5 on Tfh cells upon activation.

We found that in SIV-uninfected macaques, PD-1^hi+^ cells are predominantly negative for CCR5, though the proportion of CCR5^+^PD-1^hi+^ cells expression was significantly higher in SIV-infected macaques. Our findings are consistent with a recent report showing that GC Tfh in SIV-infected rhesus macaques include an expanded subset of cells expressing CXCR3, which is accompanied by CCR5 expression and α4β7. These GC Tfh cells, which are Th1 biased and preferentially infected, contributing disproportionately to the reservoir and contribute to hypergammaglobulinemia (7). Unexpectedly, human PD-1^hi+^ cells from HIV-uninfected subjects have a well-defined subset that exhibits low level cell surface CCR5 expression, which is further upregulated following HIV-1 infection, potentially rendering them susceptible to infection. However, the low level CCR5 expression in these cells may make this a relatively inefficient pathway for infection of Tfh.

Importantly, proviral SIV DNA was present in *ex vivo* CCR5^−^PD-1^hi+^ and CCR5^+^PD-1^hi+^ cells, at levels comparable to that in *ex vivo* CCR5^+^PD-1^int+^ cells, as well as in iPD-1^hi+^ cells derived from PD-1^int+^ cells *in vitro*. The results presented here suggest the process of infection of Tfh cells can be mediated by two possible pathways: (i) CCR5^+^PD-1^int^ cells, which represent a subset of Tfh cell precursors, are infected with SIV/HIV. Following activation these cells differentiate into viable Tfh cells carrying SIV DNA with them during this process. Some subsequently downregulate CCR5, while others maintain low CCR5 expression; or (ii) a small minority of PD-1^hi^ Tfh cells upregulate CCR5 during SIV/HIV infection, thus becoming susceptible. This latter pathway is consistent with the results of a recent study showing that purified Tfh are susceptible to infection *in vitro* with CCR5-tropic HIV-1 (18). The high rate of infection of the CCR5^−^ Tfh cells suggest that pathway (i) is the predominant pathway driving infection of Tfh as there was clear evidence of CCR5^+^PD-1^int+^ pre-Tfh downregulating CCR5 after activation. This provides a clear and plausible explanation for the infection of CCR5^−^Tfh with CCR5-using virus.

The efficient transfer of virus into pre-Tfh may be as a result of not only their CCR5 expression but because of the milieu in which they exist. The high level of cell-to-cell contact and the formation of immunological synapses may aid the transfer of the virus to these cells generated during immune responses (37). However, delineation of this process requires studies in which the architecture of the lymphoid tissue is maintained using procedures such as intravital microscopy (38). These studies may be achievable with the use of humanized murine models, if the correct lymphoid architecture and dynamics can be truly recapitulated.

A number of questions remain unanswered. Although we have shown that Tfh are preferentially infected by CCR5-using virus and have demonstrated the mechanism by which this occurs, it is unclear why they are not infected by CXCR4-using virus. Tfh and pre-Tfh are CXCR4 positive and it has been demonstrated that purified Tfh are highly susceptible to infection *in vitro* with CXCR4-tropic NL4.3 (13) and the results of our fusion assays confirm this. Despite this they are preferentially infected *in vivo* by CCR5-using virus. The relative infection efficiencies of CXCR4- and CCR5-using viruses may underlie this difference.

While the relative exclusion of cytotoxic CD8^+^ T cells from the GC partially explains the maintenance of high level of cells carrying SIV or HIV DNA and RNA in GC (35, 39, 40), the exact mechanism(s) by which Tfh precursor cells avoid direct killing by CD8^+^ T cells while in the extrafollicular area requires further delineation, but may be multifactorial (41, 42). First, it has been shown *in vivo* that SIV-specific CD8^+^ T cells are unable to effectively kill SIV-infected CD4^+^ T cells purified from extrafollicular sites of lymphoid tissue from rhesus macaques that are typical progressors (40). CTL mutational and non-mutational escape mechanisms, especially in an environment from which NK cells are normally excluded, may be another contributor. Subtle differences in the localization of these subsets within the lymphoid microenvironment while undergoing directed migration may also mean that the interactions between the Tfh precursors and CD8^+^ T cells may be infrequent.

The high frequency of infection of Tfh may be in part due to their low levels of the restriction factor SAMHD1 (43). However, this does not explain why these cells accumulate despite infection. Their high expression of the negative checkpoint regulator PD-1 combined with the relatively high expression of PD-L1 in other LN cells (44) may downregulate activation signals and increase the propensity for the development of latent infection in these cells. This is plausible since expression of PD-1 has been correlated with latent infection in PBMC in two separate studies (45, 46). Thus these two opposite modulatory tendencies may lead to both high frequency of infection but also mediate expansion of cells containing HIV DNA. The effect of infection of these cells in secondary lymphoid tissue *via* CCR5 is in distinct contrast to the massive depletion of CD4^+^ T cells in mucosa-associated lymphoid tissue. A fine dissection of the differences in these two immunological micro-environments may resolve this apparent paradox.

The findings of this study imply that CCR5 blocking therapy, such as with the approved drug Maraviroc, should be effective to protect Tfh from being infected by HIV-1 in patients with CCR5-using virus, if the active agent attains therapeutic levels in this compartment. Second, optimization of antiretroviral drug penetration into the lymphoid tissues is required. Third, suppression of specific local signals of immune activation could potentially decrease the recruitment of CCR5^+^CD4^+^ T cells into the lymphoid tissues, and the accumulation of CCR5^+^PD-1^hi+^ cells in the GC providing a mechanism for interfering with the establishment and maintenance of the viral reservoir. However, this mechanism of infection also has implications for the role of therapeutic vaccines, in reservoir clearance, as the generation of immune responses in Tfh, especially those with evidence of dysregulated function due to infection may enhance rather than restrict the reservoir (18, 20).

In summary, these findings show that Tfh cells serve as a substantial reservoir for SIV/HIV-1 infection and this reservoir is likely established and replenished by recruitment of infected precursors localized outside the GC, but within the lymphoid tissues. These findings imply that therapy should focus on blocking entry *via* CCR5, suppression of systemic immune activation, and drug penetration into the lymphoid tissues.

## Author Contributions

YX, CP, MB, KS, AA, SG-D, MR, MB, KC, AM, ST, and JZ performed experiments. SA and SK obtained lymphoid tissues from pigtail macaques. RH, RS, and KK wrote ethics protocols, recruited subjects, and performed surgery. YX, BA, PG, DC, AM, AK, and JZ conceived and supervised experiments. YX, PG, SK, AM, AK, and JZ wrote manuscript.

## Conflict of Interest Statement

The authors declare that the research was conducted in the absence of any commercial or financial relationships that could be construed as a potential conflict of interest.
