# Supplementary material for: HIV-1 and SIV Predominantly Use CCR5 Expressed on a Precursor Population to Establish Infection in T Follicular Helper Cells
Source: Front Immunol. 2017 Apr 21;8:376. doi: 10.3389/fimmu.2017.00376 (PMC5399036; doi:10.3389/fimmu.2017.00376)
Supplement: Supplementary file 1 [file table_1.docx]

**Supplementary figures**

**Fig S1**


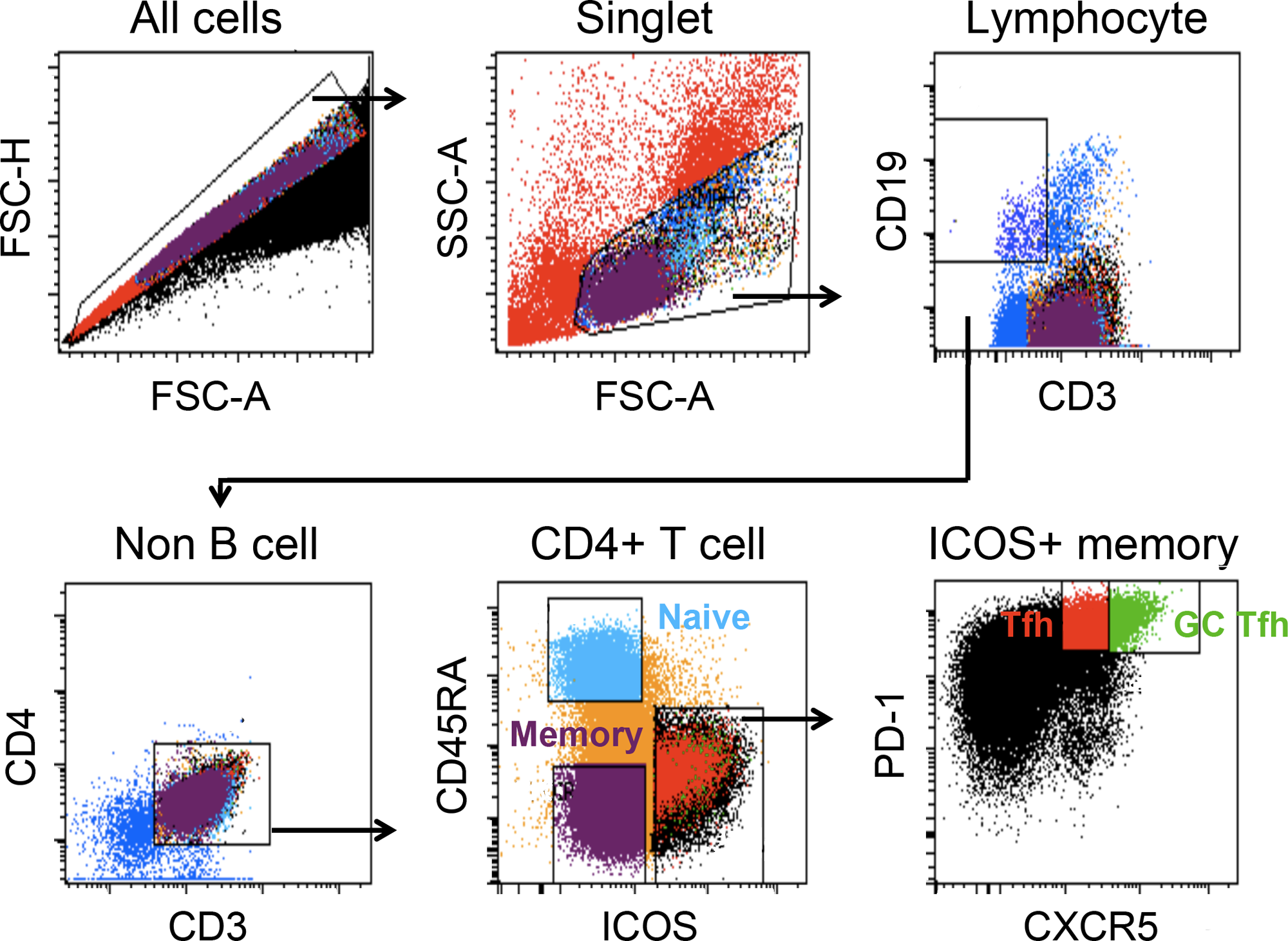


**Fig S1. Sorting strategy for single cell suspensions of human splenic lymphocytes.**

Sorting resulted in purified subsets of naïve (CD45RA^+^ICOS^-^, blue) and resting memory (CD45RA^-^ICOS^-^, purple) cells, and two subsets of Tfh defined as: (i) CXCR5^int+^ Tfh (CD45RA^-^ICOS^+^PD-1^hi+^CXCR5^int+^, red); and (ii) CXCR5^hi+^ germinal center (GC) Tfh (CD45RA^-^ ICOS^+^ PD-1^hi+^CXCR5^hi+^ , green).

**Fig S2**


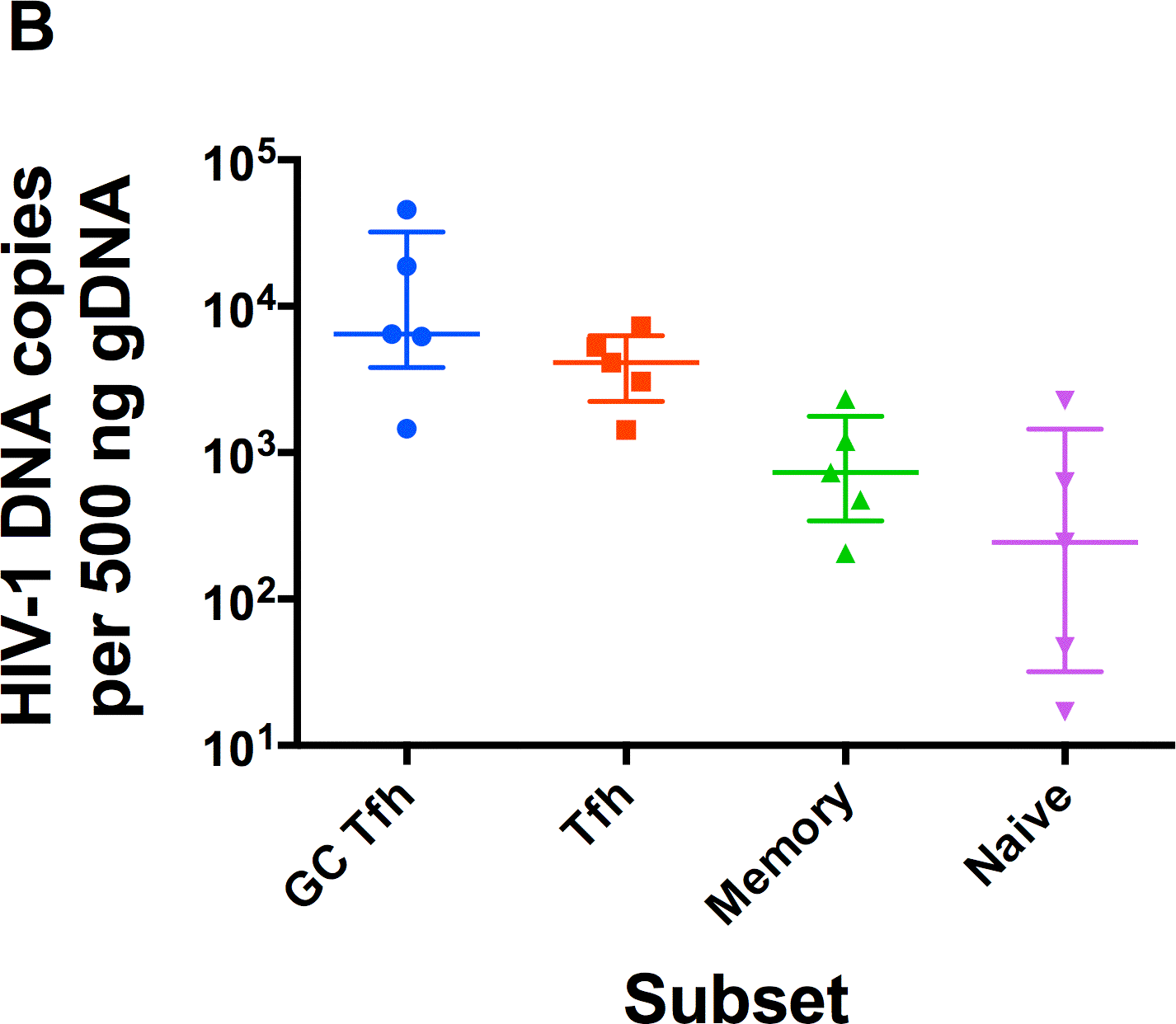


**Fig S2. HIV-1 *gag* DNA levels in sorted CD4^+^ T cell populations.**

Summarized data of HIV-1 DNA copies per 500 ng gDNA in the four sorted CD4^+^ T cell subsets as shown in Fig S1. Error bars indicate median and interquartile range.

**Fig S3**


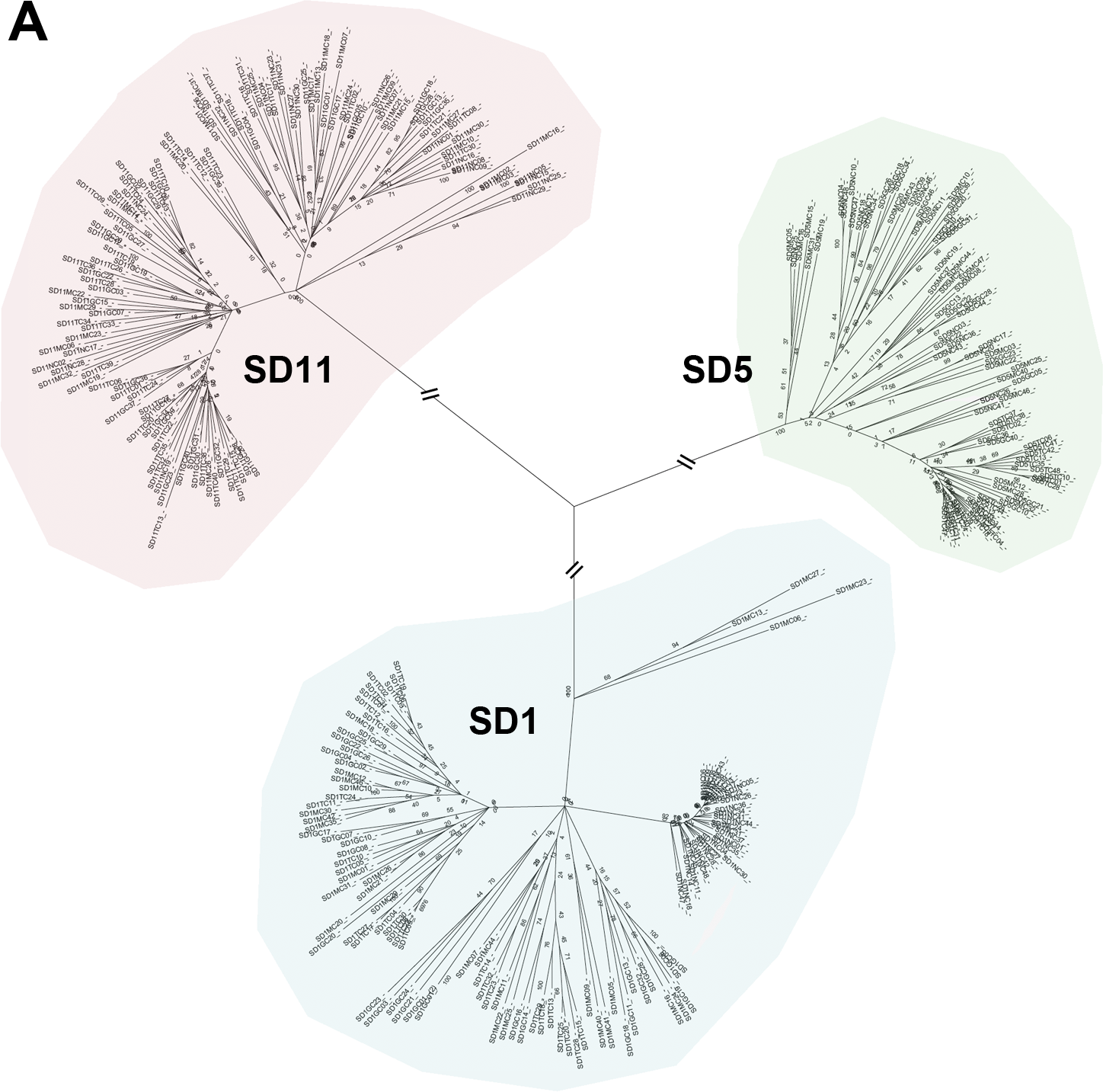


**Fig S3. HIV Sequences from each patient form distinct clusters.**

Bootstrapped consensus tree constructed with clones from all three patients (pale red: SD11, pale blue: SD5, pale green: SD1). The tree inferred from 1000 replicates was constructed using the Neighbor-Joining method with the bootstrap values shown at the branches.

**Fig S4**


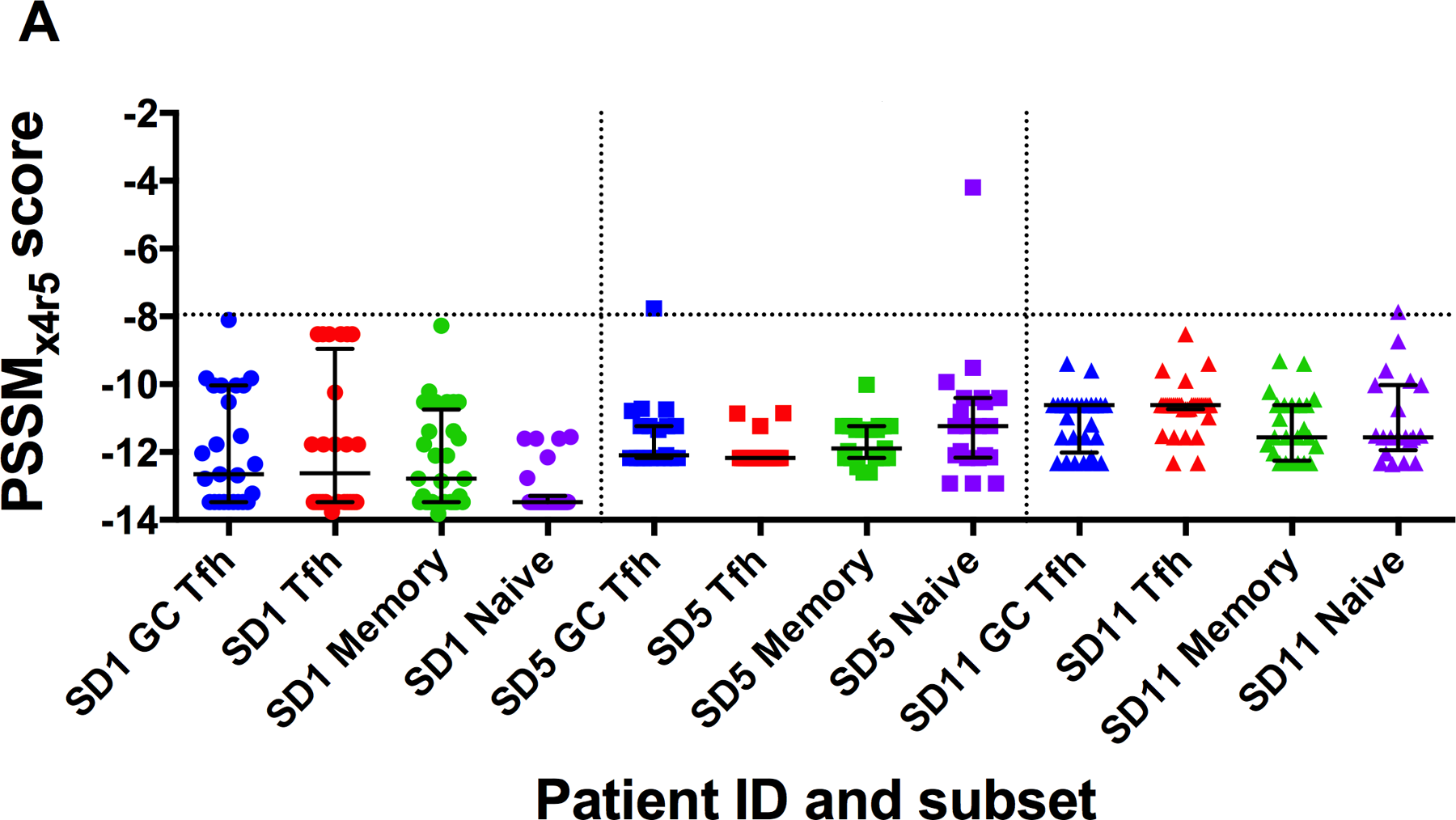


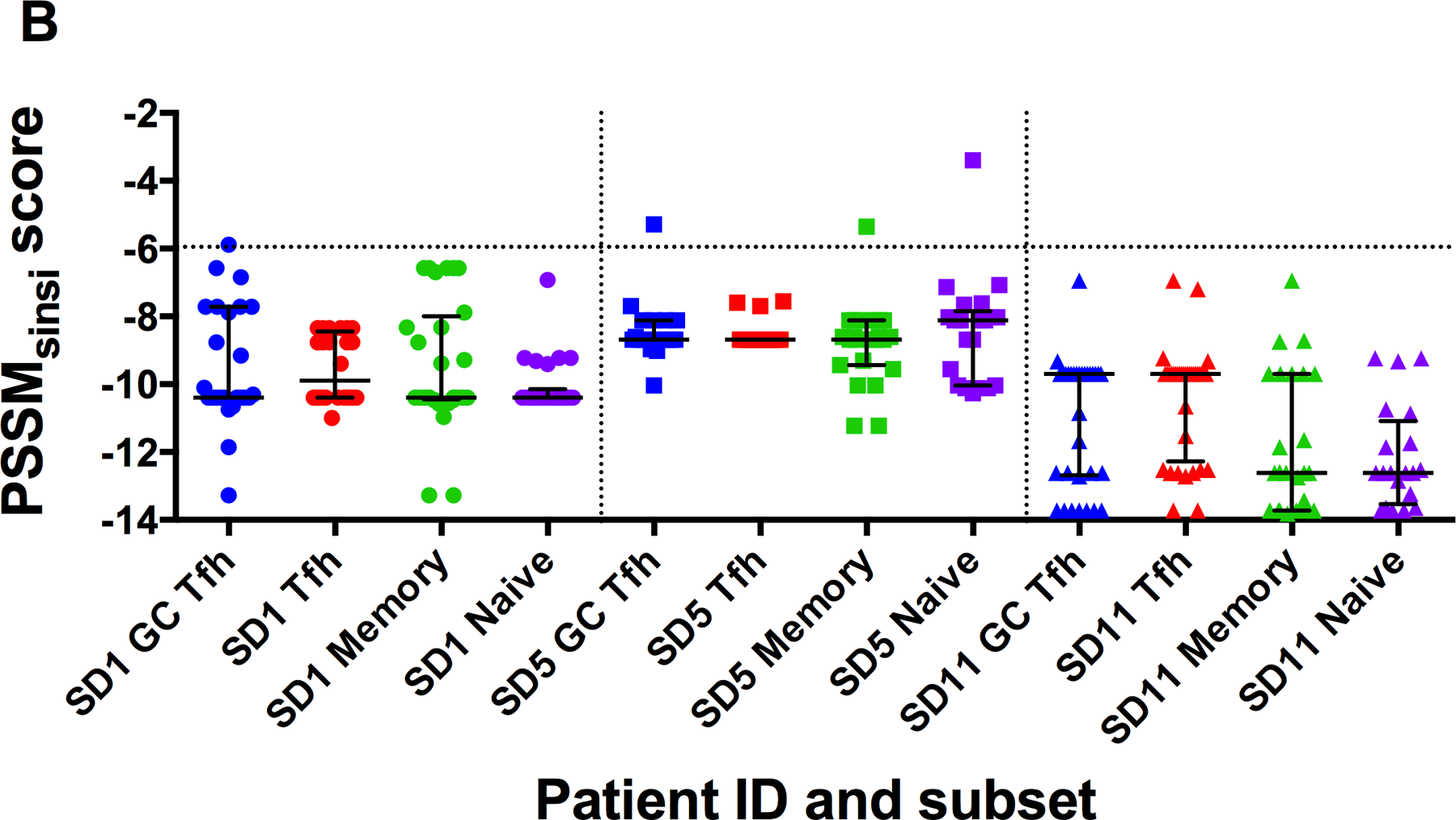


**Fig S4. Env sequences from each cell subset from each patient have low PSSMX4r5 and PSSMsinsi scores indicating a bias towards CCR5 usage.**

Cut-offs for PSSMX4r5 (-8) and for PSSMsinsi (-6) shown as dotted lines. Results below the cut-off are consistent with a CCR5-using virus (A) or non-syncytium (NSI) (B) respectively.

**Fig S5**

**
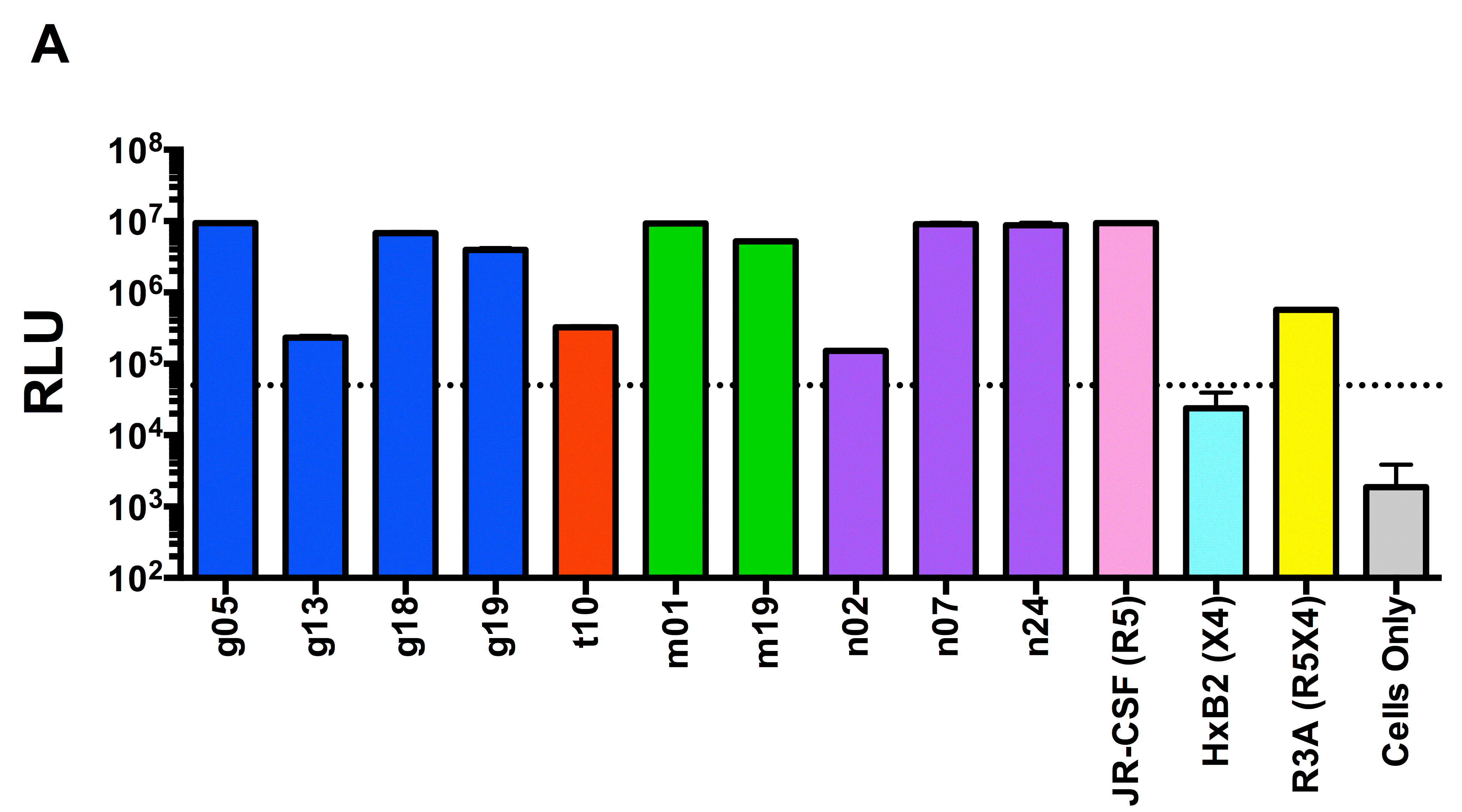
**

**
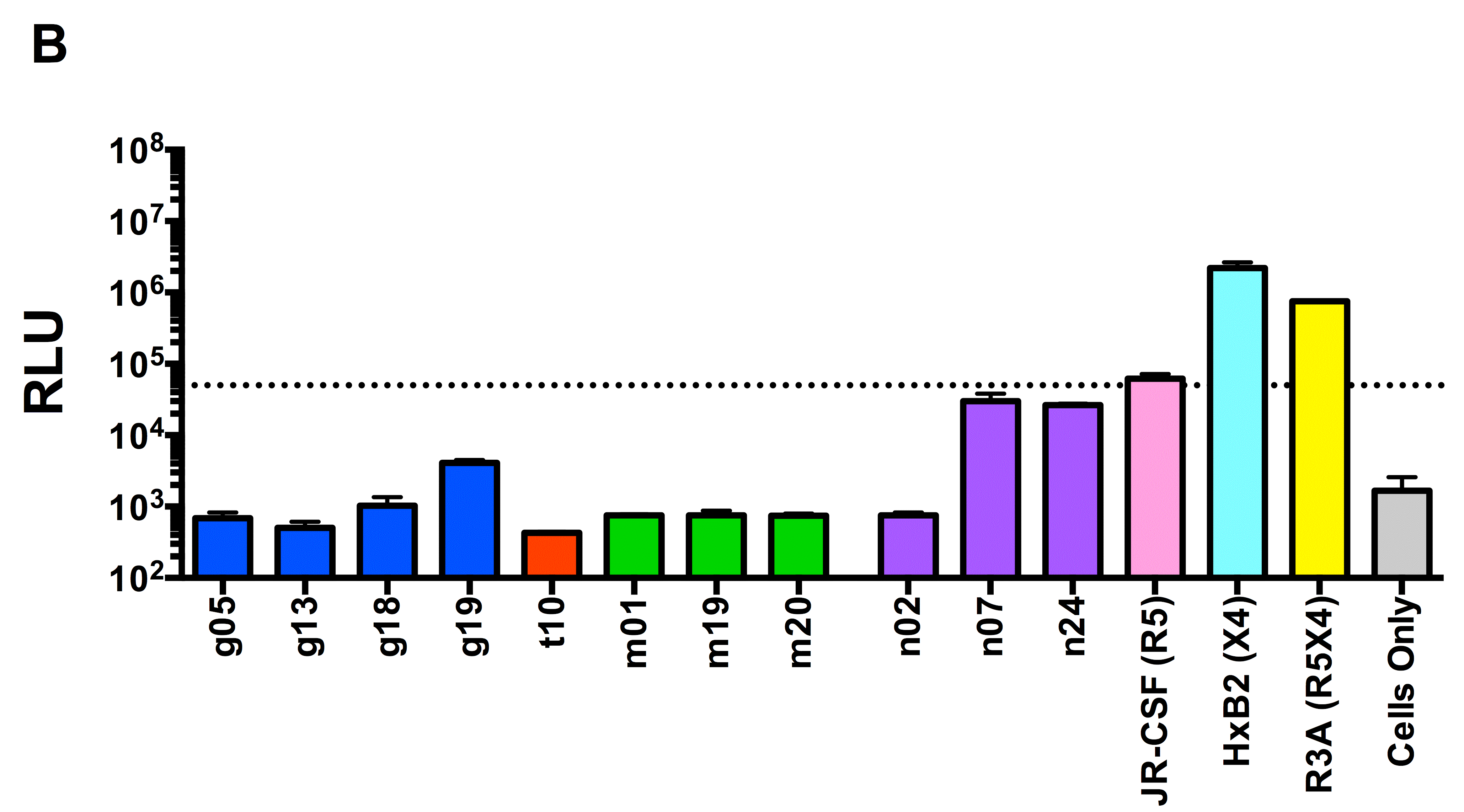
**

Fig S5. HIV-1 envelope clones from different CD4^+^ T cell subsets were CCR5-using.

Coreceptor usage of pseudovirus containing HIV-1 envelope gp120 cloned from patient’s provirus was assessed by phenotypic assay. Pseudoviruses containing HIV-1 laboratory strains typical of CCR5-using (JR-CSF, pink), CXCR4-using (HXB2, cyan) and dual tropic (R3A, yellow) were used as controls. The efficiency of viral entry into (**A**) NP2-CD4/CCR5 or (**B**) NP2-CD4/CXCR4 cells is indicated by the luciferase signal. Cut-off was set to 5×10^4^ RLU, indicated by the horizontal dotted line.

**Fig S6**

**
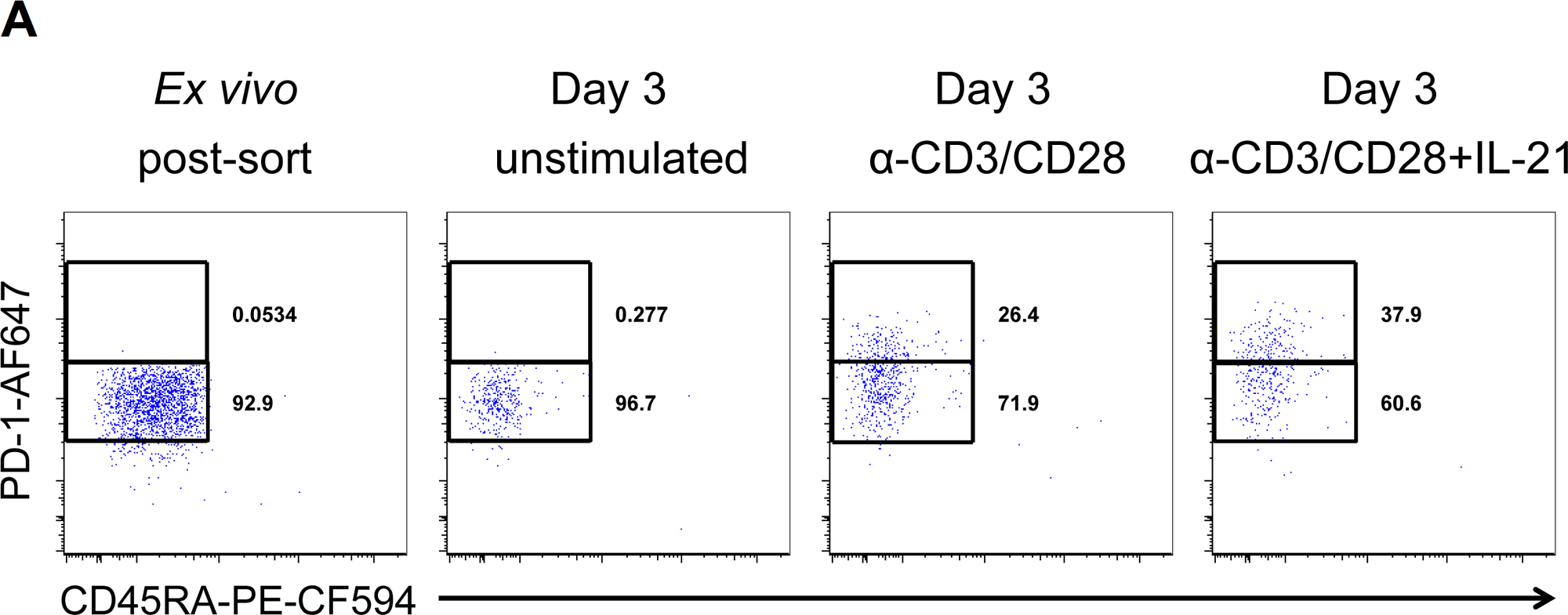
**

**
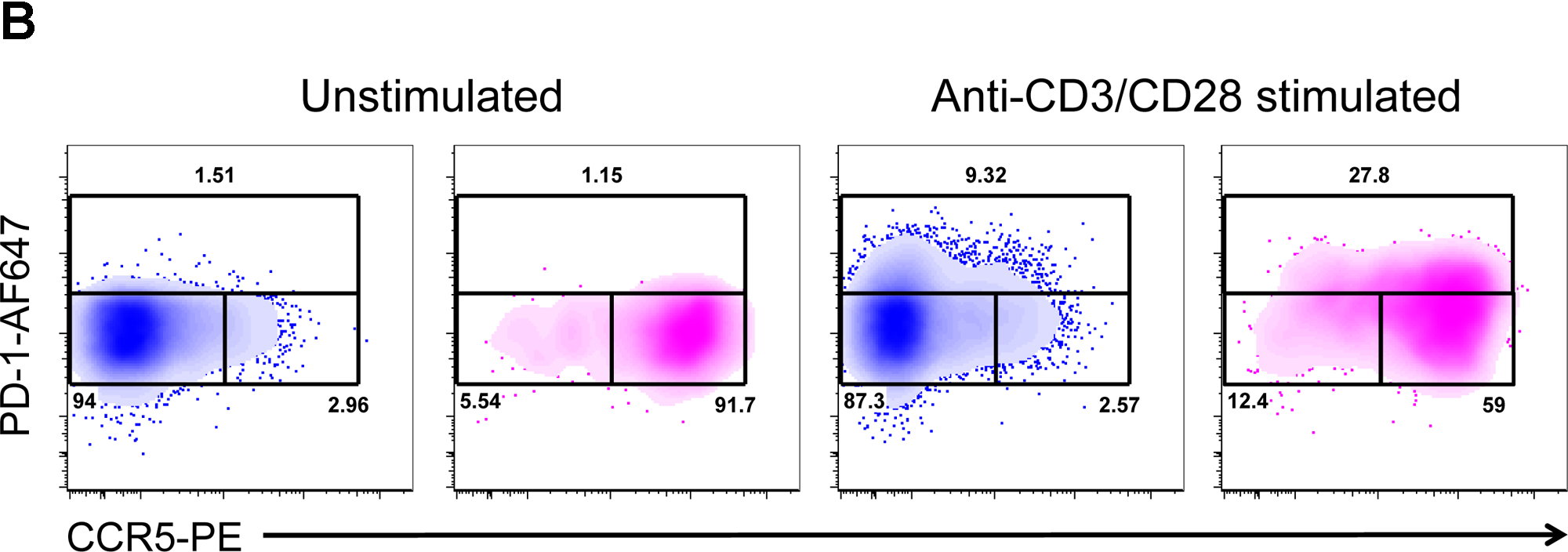
**

**Fig S6. Representative plots of effects of stimulating purified CD4^+^CD45RA^-^PD-1^int+^ T cells through TCR: up-regulation of PD-1 and CCR5 expression.**

**Fig S7**

**
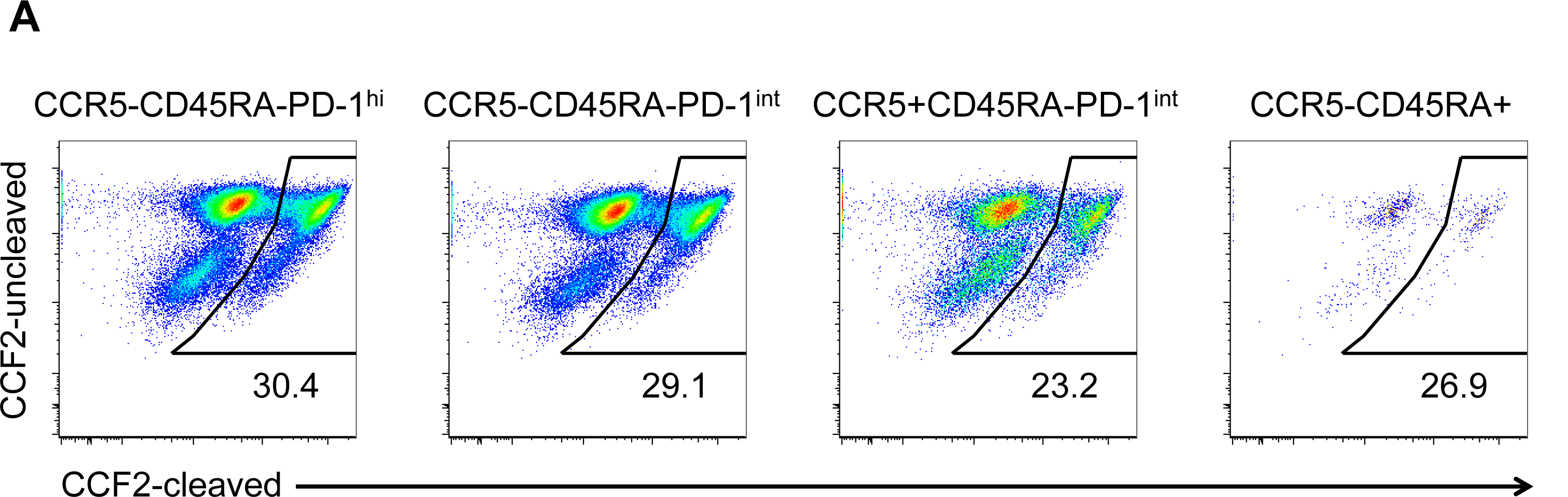
**

**
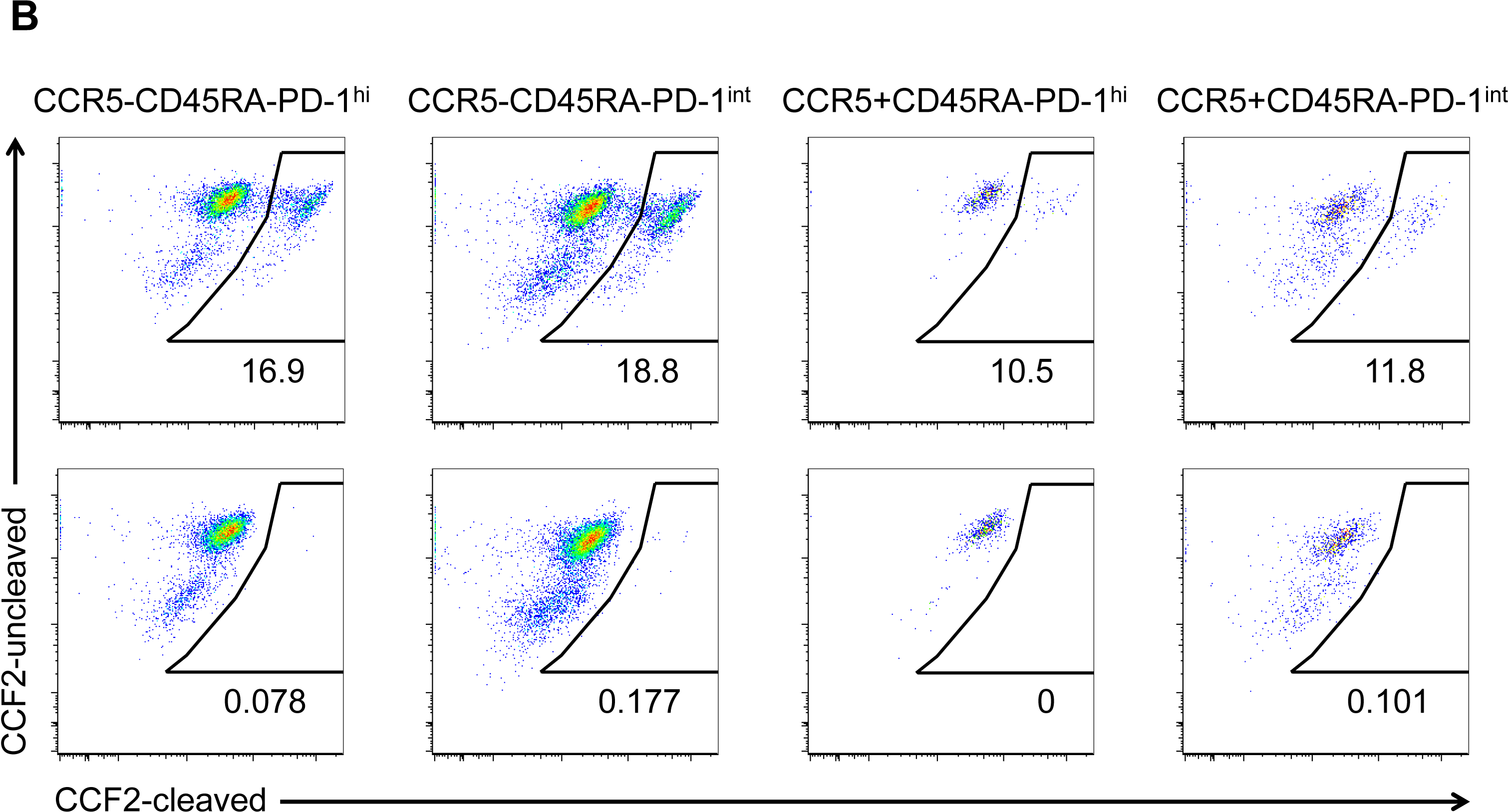
**

**
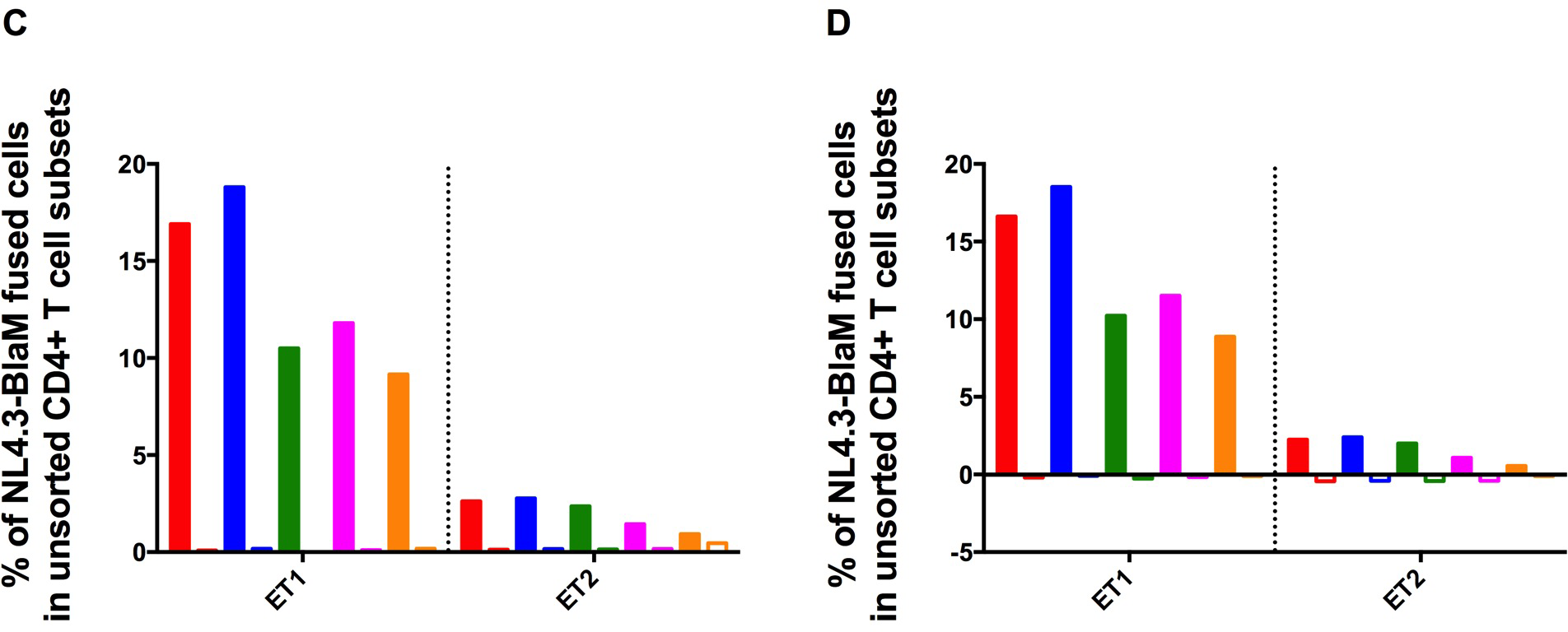
**

**Fig S7. Fusion of HIV NL-4.3 using human CD4^+^ T cell subsets derived from tonsils.**

**A.** Representative flow plots showing fusion of NL4.3-BlaM into purified CD4+ T cell subsets.

**B.** Representative flow plots showing fusion of NL4.3-BlaM with phenotypically defined subsets within bulk unsorted cell populations.

**C.** Column bars showing the percentage of NL4.3-BlaM fused cells in CD4+ T cell subsets from unsorted samples (red: CCR5^-^CD45RA^-^PD-1^hi+^, blue: CCR5^-^CD45RA^-^PD-1^int+^, green: CCR5^+^CD45RA^-^PD-1^hi+^, magenta: CCR5^+^CD45RA^-^PD-1^int+^, orange: CCR5^-^ CD45RA^+^), in the absence (filled) and presence of AMD3100 (clear).

**Supplementary Tables**

**Table S1. Pigtail macaques included in this study.**

| **Monkey ID** | **SIV strain** | **Vaccine received** | **Days post infection** | **^a^ Plasma viral load (log10 copy/ml)** | **Peripheral CD4+ T cell percentage of CD3+ T cells on the day of biopsy taken (%)** | **Peripheral CD4+ T cell count as percentage of baseline (%)** |
| --- | --- | --- | --- | --- | --- | --- |
| **5873** | SIVmac251 | Control | Euthanized on day 274 | 6.0 on day 231 | 52.2 | 84.7 |
| **C3751** | SIVmac251 | Control | ILN taken on day 14 | 8.2 | 39.9 | 81.3 |
| **45418** | SIVmac251 | Flu-SIV | ILN taken on day 70 | 4.0 on day 56 | 50.1 | 87.1 |
| **B0433** | SIVmac251 | Flu-SIV | MLN taken on day 6 | 3.1 | 52.5 | 112.7 |
| **B0526** | SIVmac251 | Flu-SIV | Euthanized on day 196 | 6.0 on day 168 | 24.3 | 63.6 |
| **B0527** | SIVmac251 | Flu-SIV | Euthanized on day 14 | 3.1 | 50.4 | 108.2 |
| **C3754** | SIVmac251 | None | ILN taken on day 70 | 6.2 on day 56 | 44.0 | 62.8 |
|  |  |  | Euthanized on day 224 | 7.4 on day 182 | 35.8 | 46.2 |
| **19530** | SIVmac251 | None | ILN taken on day -35 | N/A | 59.6 | N/A |
|  |  |  | ILN taken on day 70 | N/D | 43.3 | 89.0 |
| **6273** | SIVmac239 | None | ILN taken on day 8 | <3.1 | 58.9 | 95.9 |
|  |  |  | ILN taken on day 28 | 6.6 | 36.3 | 59.1 |
| **6870** | SHIVSF162P3 (CCR5) | HIV-1 Gp140 | ILN taken on day 14 | 6.8 | 56.6 | 90.1 |
| **19340** | SIVmac239 | None | ILN taken on day 168 | 5.6 | 25.9 | 5.96 |
| **36142** | SIVmac239 | None | ILN taken on day 194 | 5.6 | 24.5 | 3.88 |
| **7476** | SIVmac239 | None | ILN taken on day 28 | 5.3 | 40.6 | 87.7 |
|  |  |  | Euthanized on day 237 | N/A | 27.7 | N/A |
| **5504** | SIVmac239 | None | ILN taken on day 28 | 5.4 | 39.5 | 85.5 |
|  |  |  | Euthanized on day 77 | 6.3 | 41.4 | 82.6 |

N/A: Not available

N/D: Not done

**Table S2. Information of primers used in this study.**

| **Primer** | **Sequence*** | **Optimal annealing temperature** | **Amplicon size** | **Reference** |
| --- | --- | --- | --- | --- |
| β-actin-F | 5’-TCACCCACACTGTGCCCATCTACGA-3’ | 60°C | 295 bp | (1) |
| β-actin-R | 5’-CAGCGGAACCGCTCATTGCCAATGG-3’ |  |  |  |
| *ccr5*-F | 5’-GTCCCCTTCTGGGCTCACTAT-3’ | 64°C | 72 bp | (2) |
| *ccr5*-R | 5’-CCCTGTCAAGAGTTGACACATTGTA-3’ |  |  |  |
| *gag_SIV_*-F | 5’-AATTAGATAGATTTGGATTAGCAGAAAGC-3’ | 60°C | 142 bp | (3) |
| *gag_SIV_*-R | 5’-CACCAGATGACGCAGACAGTATTAT-3’ |  |  |  |
| mf299 | 5’-GCACTTTAAATTTTCCCATTAGTCCTA-3’ | 60°C | 127 bp | (4) |
| mf302 | 5’-CAAATTTCTACTAATGCTTTTATTTTTTC-3’ |  |  |  |
| ABI- β-actin-F | 5’-CGGAACCGTCATTGCC-3’ | 60°C | 289 bp | (5) |
| ABI- β- actin-R | 5’-ACCCACACTGTGCCCATCTA-3’ |  |  |  |
| E00F | 5’-AGCAGAAGACAGTGGCAATGA-3’ | 55°C | 1.6 kb, variable | Designed by Dr. Suzuki, K. |
| CO602N | 5’-GCCCATAGTGCTTCCTGCTGCTCCCAAGAACC-3’ |  |  |  |
| E20F | 5’-ACATGCCTGTGTACCCAC-3’ | 59°C | 1.2 kb, variable | Designed by Dr. Suzuki, K. |
| CD4R | 5’-TATAATTCACTTCTCCAATTGTCC-3’ |  |  |  |
| M-*actb*-F | 5’-AGAGCTACGAGCTGCCCGAT-3’ | 60 °C | 114 bp | Modified from (6) |
| M-*actb*-R | 5’-CGTGGATGCCACAGGACT-3’ |  |  |  |
| HM-*tbx21*-F | 5’-CACCTGTTGTGGTCCAAGTTT-3’ | 60 °C | 84 bp | (6) |
| HM-*tbx21*-R | 5’-TGACAGGAATGGGAACATCC-3’ |  |  |  |
| M-*gata3*-F | 5’-AACTGTCAGACCACCACGACCACAC-3’ | 60 °C | 130 bp | Modified from (6) |
| M-*gata3*-R | 5’-GGATGCCTTCCTTCTTCATGGTCAGG-3’ |  |  |  |
| M-*rorc*-F | 5’-TGAGAAGGACAGGGAGCCAA-3’ | 60 °C | 85 bp | Designed by Dr. Phetsouphanh, C. |
| M-*rorc*-R | 5’-CCACAGATTTTGCAAGGGATCA-3’ |  |  |  |
| M-*bcl6*-F | 5’-CGAATCCACACGGGAGAGAAA-3’ | 60 °C | 131 bp | Modified from (6) |
| M-*bcl6*-R | 5’-ACGCGGTATTGCACCTTG-3’ |  |  |  |
| HM-*foxp3*-F | 5’-GACCAAGGCTTCATCTGTGG-3’ | 60 °C | 125 bp | (6) |
| HM-*foxp3*-R | 5’-CAGCAAACAGGCTGTCAGG-3’ |  |  |  |
| HIV-*gag*-F | 5’-AGTGGGGGGACATCAAGCAGCC-3’ | 60 °C | 155 bp | (7) |
| HIV-*gag*-R | 5’-TACTAGTAGTTCCTGCTATGTCACTTCC-3’ |  |  |  |
| Env-*Kpn*I | 5’-GTCTATTATGG**GGTACC**TGTGTGG-3’ | 60 °C | ~119 bp | (8) |
| E20R | 5’-GTGGGTACACAGGCATGT-3’ |  |  |  |
| CD4F | 5’-GGACAATTGGAGAAGTGAATTATA-3’ | 60 °C | ~833 bp | (8) |
| Env-*Bam*HI | 5’-GCTAA**GGATCC**GTTCACTAATCGT-3’ |  |  |  |

* Locked nucleic acid (LNA) bases are denoted in [] with +. All probes are dual labelled with 6-FAM (6-carboxyfluorescence) at the 5’- end and BHQ1 (black hole quencher 1) at the 3’- end.

Letters in bold indicate restriction sites.

Table S3. Percentage of predicted CXCR4 variants predicted with PhenoSeq-B and Geno2Pheno_[coreceptor]_ at cut-off of 10% and the concordance between these two algorithms.

| **Patient** | **Percentage of predicted CXCR4 variants** | | **Consistency** | **Fisher’s exact *p* value** |
| --- | --- | --- | --- | --- |
|  | **PhenoSeq-B** | **G2P at cut-off of 10%** |  |  |
| **SD1** | 23.3% | 24.27% | 83.50% | 1.00 |
| **SD5** | 5.75% | 5.75% | 93.10% | 1.00 |
| **SD11** | 4.46% | 4.46% | 96.43% | 1.00 |

Table S4. Sources of human lymphoid tissue from HIV uninfected patients.

| **Sample ID** | **Tissue type** | **Note** |
| --- | --- | --- |
| **ELN** | Excised LN | Diagnostic material |
| **ES** | Excised spleen | From patient with thrombocytopaenia 2° to hypersplenism |
| **ET1** | Excised tonsil | For clinical management |
| **ET2** | Excised tonsil | For clinical management |
| **FNB1** | ILN fine needle biopsy | From healthy staff control |
| **FNB2** | ILN fine needle biopsy | From healthy staff control |
| **FNB3** | ILN fine needle biopsy | From healthy staff control |

**Reference:**

1. Gelmini S, Orlando C, Sestini R, Vona G, Pinzani P, Ruocco L, et al. Quantitative polymerase chain reaction-based homogeneous assay with fluorogenic probes to measure c-erbB-2 oncogene amplification. *Clin Chem* (1997) **43**:752-8. Epub 1997/05/01. PubMed PMID: 9166227.

2. Mattapallil JJ, Douek DC, Hill B, Nishimura Y, Martin M, Roederer M. Massive infection and loss of memory CD4+ T cells in multiple tissues during acute SIV infection. *Nature* (2005) **434**:1093-7. Epub 2005/03/29. doi: nature03501 [pii] 10.1038/nature03501. PubMed PMID: 15793563.

3. Shehu-Xhilaga M, Kent S, Batten J, Ellis S, Van der Meulen J, O'Bryan M, et al. The testis and epididymis are productively infected by SIV and SHIV in juvenile macaques during the post-acute stage of infection. *Retrovirology* (2007) **4**:7. Epub 2007/02/03. doi: 1742-4690-4-7 [pii] 10.1186/1742-4690-4-7. PubMed PMID: 17266752; PubMed Central PMCID: PMC1805449.

4. Althaus CF, Gianella S, Rieder P, von Wyl V, Kouyos RD, Niederost B, et al. Rational design of HIV-1 fluorescent hydrolysis probes considering phylogenetic variation and probe performance. *J Virol Methods* (2010) **165**:151-60. Epub 2010/02/02. doi: 10.1016/j.jviromet.2010.01.012. PubMed PMID: 20116399.

5. du Breuil RM, Patel JM, Mendelow BV. Quantitation of beta-actin-specific mRNA transcripts using xeno-competitive PCR. *PCR Methods Appl* (1993) **3**:57-9. Epub 1993/08/01. PubMed PMID: 8220185.

6. Phetsouphanh C, Xu Y, Amin J, Seddiki N, Procopio F, Sekaly RP, et al. Characterization of transcription factor phenotypes within antigen-specific CD4+ T cells using qualitative multiplex single-cell RT-PCR. *PLoS One* (2013) **8**:e74946. Epub 2013/10/15. doi: 10.1371/journal.pone.0074946. PubMed PMID: 24124462; PubMed Central PMCID: PMC3790772.

7. Suzuki K, Shijuuku T, Fukamachi T, Zaunders J, Guillemin G, Cooper D, et al. Prolonged transcriptional silencing and CpG methylation induced by siRNAs targeted to the HIV-1 promoter region. *J RNAi Gene Silencing* (2005) **1**:66-78. Epub 2005/01/01. PubMed PMID: 19771207; PubMed Central PMCID: PMC2737205.

8. Ohagen A, Devitt A, Kunstman KJ, Gorry PR, Rose PP, Korber B, et al. Genetic and functional analysis of full-length human immunodeficiency virus type 1 env genes derived from brain and blood of patients with AIDS. *J Virol* (2003) **77**:12336-45. Epub 2003/10/29. PubMed PMID: 14581570; PubMed Central PMCID: PMC254258.
